# Polymorphism and Thermally Induced Structural Transformation in Semiconducting Cd(II) Coordination Polymers

**DOI:** 10.1021/acs.inorgchem.5c02821

**Published:** 2025-09-02

**Authors:** Ryohei Akiyoshi, Honoka Motokawa, Asuka Nishibe, Atsuki Yanai, Takanori Nakane, Akihiro Kawamoto, Genji Kurisu, Akinori Saeki, Daichi Eguchi, Naoto Tamai, Yuki Mori, Shogo Kawaguchi, Kazuyoshi Ogasawara, Daisuke Tanaka

**Affiliations:** † Department of Chemistry, School of Science, 12907 Kwansei Gakuin University, 1 Gakuen Uegahara, Sanda, Hyogo 669-1330, Japan; ‡ Institute for Protein Research, 13013 The University of Osaka, 3-2 Yamadaoka, Suita, Osaka 565-0871, Japan; § JEOL YOKOGUSHI Research Alliance Laboratories, Graduate School of Frontier Biosciences, The University of Osaka, 1-3 Yamadaoka, Suita, Osaka 565-0871, Japan; ∥ Department of Applied Chemistry, Graduate School of Engineering, The University of Osaka, 2-1 Yamadaoka, Suita, Osaka 565-0871, Japan; ⊥ 133704 Japan Synchrotron Radiation Research Institute (JASRI), 1-1-1 Kouto, Sayo-cho, Sayo-gun, Hyogo 679-5198, Japan

## Abstract

Three polymorphs of a semiconducting Cd­(II)-thiolate coordination polymer with the formula [Cd­(2-SNPh)_2_]_*n*_ (2-HSNPh = 2-naphthalenethiol) have been reported. Single-crystal X-ray structural analysis revealed that **KGF-51** adopts a one-dimensional (1D) structure composed of single-strand (−Cd–S−)_*n*_ ladder chains, whereas **KGF-71** adopts a 1D architecture with double-strand (−Cd–S−)_*n*_ chains assembled through S···S interactions. In contrast, microcrystal electron diffraction analysis demonstrated that **KGF-79**, obtained through thermal treatment of **KGF-51** and **KGF-71**, exhibits the formation of a 1D structure comprising triple-strand (−Cd–S−)_*n*_ chains, also formed via S···S interactions. This thermally induced, irreversible structural transformation to **KGF-79** is accompanied by a transition from a nonpolar structure to a polar structure, leading to the emergence of second harmonic generation activity. Furthermore, time-resolved microwave conductivity measurements and first-principles calculations demonstrated that all three polymorphs exhibit photoconductivity, which is attributed to charge transport along the inorganic (−Cd–S−)_*n*_ chains via a hopping mechanism.

## Graphical abstract

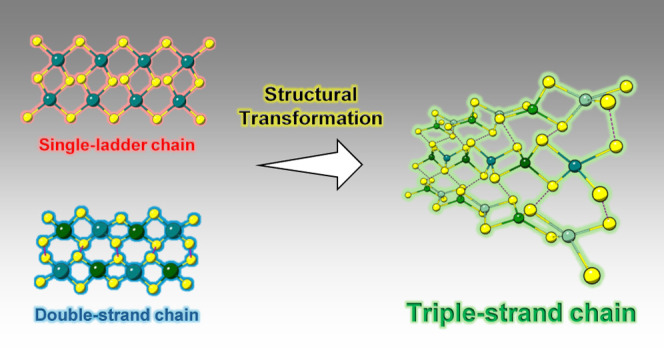

## Introduction

Coordination polymers containing sulfur coordination atoms (namely, S-CPs)^1–6^ have attracted significant attention in recent years due to their potential applications in photocatalysis,^7–12^ chemiresistive sensors,^13–15^ and optoelectronic devices.^16–18^ In particular, S-CPs with metal–sulfur (−M–S−)_*n*_ networks are of significant interest owing to their unique optoelectronic properties including a narrow band gap energy and excellent charge mobility.^19–21^ The optoelectronic properties of these materials are primarily governed by the network structure of the inorganic (−M–S−)_*n*_ moiety formed therein, which can be precisely tuned through ligand design.^22,23^

To elucidate the relationship between the (–M–S–)_*n*_ network and their optoelectronic properties, researchers have systematically synthesized S-CPs using benzenethiol derivatives (HSPhX, X = substituents). These derivatives are highly tunable, making them ideal for studying structure–property relationships. Among d^10^ coinage metal-based S-CPs, the type and position of substituents on the HSPh skeleton significantly influence the dimensionality of the (−M–S−)_*n*_ network, consequently impacting their optoelectronic behavior.^24–29^ For example, Demessence et al. synthesized Au­(I)-SPhX with various functional groups and reported corresponding structural transformation from a one-dimensional (1D) double helical chain of [Au­(SPh)]_*n*_ to two-dimensional (2D) lamella of [Au­(*p*-SPhCO_2_H)]_*n*_, that in turn lead to an increase in the photoluminescence quantum yield.^26^ Similarly, a systematic study on Pb­(II) S-CPs using HSPhX (X = OCH_3_, SCH_3_, COOH, COOCH_3_) ligands revealed that variations in the dimensionality of the (−Pb–S−)_*n*_ network lead to significant changes in the band gap energy and photoconductivity.^22,23^ These findings highlight the importance of engineering of (−M–S−)_*n*_ networks for precisely tuning the optoelectronic properties of S-CPs. However, to accurately discuss the effect of (−M–S−)_*n*_ networks on the resulting properties, comparative studies involving S-CPs polymorphs with the same chemical compositions but with different inorganic (−M–S−)_*n*_ networks are a critical requirement.

Recently, we reported the polymorphism of a 2D semiconductive Pb­(II) S-CP, wherein thermal treatment induces a structural transformation between polymorphs, leading to variations in the (−Pb–S−)_*n*_ network, which in turn alters the photoconductivity.^30^ Investigating polymorphisms and structural transformations in S-CPs provides valuable insights into the detailed relationship between the network structure and electronic properties, yet reports on polymorphisms of S-CPs remain scarce except Pb­(II) S-CPs.^30,31^

In this study, Cd­(II) ions were selected as a metal center since Cd­(II) S-CPs with HSPh derivative ligands adopt diverse crystal structures depending on substituent species, implying their potential for polymorphism.^29,32,33^ The variation in semiconducting properties, arising from the differences in the assembly of the (−Cd–S−)_*n*_ network structure, also motivated us to explore the crystal polymorphs of the Cd­(II) S-CPs. Herein, we report three distinct polymorphs of Cd­(II) S-CPs with the formula [Cd­(2-SNPh)_2_] (2-HSNPh = 2-naphthalenethiol). **KGF-51** features a nonpolar 1D structure with single-strand (−Cd–S−)_*n*_ ladder chains, while **KGF-71** adopts a nonpolar 1D structure composed of double-strand (−Cd–S−)_*n*_ chains assembled via S···S interactions. Notably, both **KGF-51** and **KGF-71** irreversibly transform to **KGF-79** upon thermal treatment, yielding a polar 1D architecture with triple-strand (−Cd–S−)_*n*_ chains formed through S···S interactions that exhibits second harmonic generation (SHG) activity (Scheme 1). Time-resolved microwave conductivity (TRMC) measurements and first-principles calculations revealed that all three polymorphs exhibit photoconductivity originating from inorganic (−Cd–S−)_*n*_ chains.

**Scheme 1:**
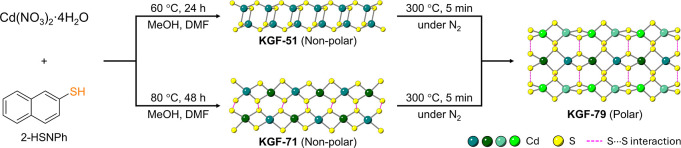
Schematic for the Synthesis and Structures of **KGF-51**, **KGF-71**, and **KGF-79**. The synthetic procedures for **KGF-51** and **KGF-71** shown above correspond to the preparation of their pure bulk powders

## Results and Discussion

Colorless needle crystals of **KGF-51** were prepared by solvothermal treatment using EtOH/DMF solvents (v:v = 1:1) at 80 °C (Figure S1a). The crystal structure of **KGF-51** was determined by a single-crystal X-ray diffraction (SCXRD) measurement at 150 K. **KGF-51** crystallized in the triclinic *P*1̅ space group (nonpolar space group) (Table S1). The asymmetric unit of **KGF-51** consists of one Cd­(II) ion and two deprotonated 2-SNPh^–^ ligands (Figure 1a). The Cd­(II) center affords a [CdS_4_]-based tetrahedral coordination geometry (Figure S2a). The Cd–S bond distances are in the range of 2.5002(7)–2.5973(7) Å, which are consistent with those of previously reported Cd­(II) S-CPs.^29^ Both the S1 and S2 atoms of 2-SNPh^–^ ligands act as μ_2_-S-bridging atoms. The S1 atom connected two Cd­(II) ions that formed dinuclear units, whereas the S2 atom bridged adjacent dinuclear units. Consequently, **KGF-51** formed a 1D architecture consisting of an inorganic (−Cd–S−)_*n*_ ladder chain along the *a*-axis (Figures 1b,c and S3a). The resulting 1D chains were further assembled by weak van der Waals (vdW) interactions (Figure S4a).

**Figure 1:**
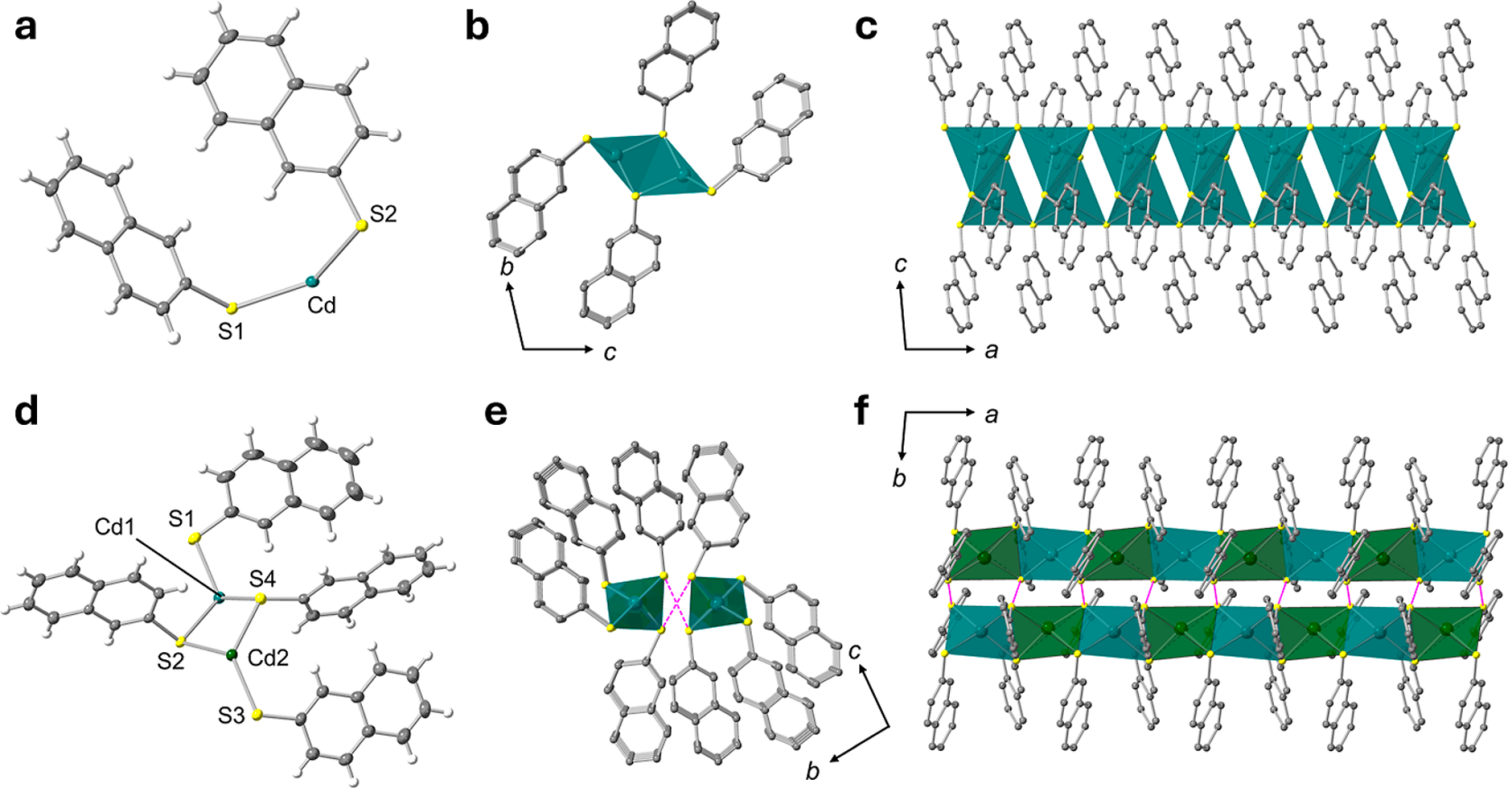
SCXRD analysis of **KGF-51** and **KGF-71**. Asymmetric units of (a) **KGF-51** and (d) **KGF-71**. Ellipsoids were presented in 50% probability. 1D chain structures of (b,c) **KGF-51** and (e,f) **KGF-71**. Color code: Cd, green; S, yellow; C, gray; H, white. Purple lines indicate the S···S interactions between (−Cd–S−)_*n*_ chains.

Single crystals of **KGF-71** were obtained as colorless needle crystals by a solvothermal reaction under MeOH/DMF solvents (v:v = 1:1) at 80 °C (Figure S1b). The SCXRD measurement at 150 K revealed that **KGF-71** crystallized in the triclinic *P*1̅ space group (nonpolar space group) (Table S1). Unlike **KGF-51**, the asymmetric unit of **KGF-71** consists of two Cd­(II) ions and four 2-SNPh^–^ ligands (Figure 1d). Similar to **KGF-51**, both Cd1 and Cd2 adopt [CdS_4_] tetrahedron coordination geometries (Figures S2b). The Cd–S bond distances range from 2.5286(8) to 2.6043(7) Å, consistent with that of **KGF-51**. The S atoms of **KGF-71** are μ_2_-S atoms, alternatively linking Cd1 and Cd2 ions. Consequently, **KGF-71** features a 1D structure with inorganic (−Cd–S−)_*n*_ chains whose Cd1 and Cd2 are alternatively aligned (Figure S3b). Notably, two of these 1D chains were assembled through interchain S···S interactions, thus forming a double-stranded chain structure (Figure 2e,f). The S···S distances are 3.045(1) and 3.1006(9) Å that are comparable to previously reported Cd­(II) S-CPs.^29^ The resultant double-strand chains were further assembled by the vdW interaction (Figure S4b).

**Figure 2:**
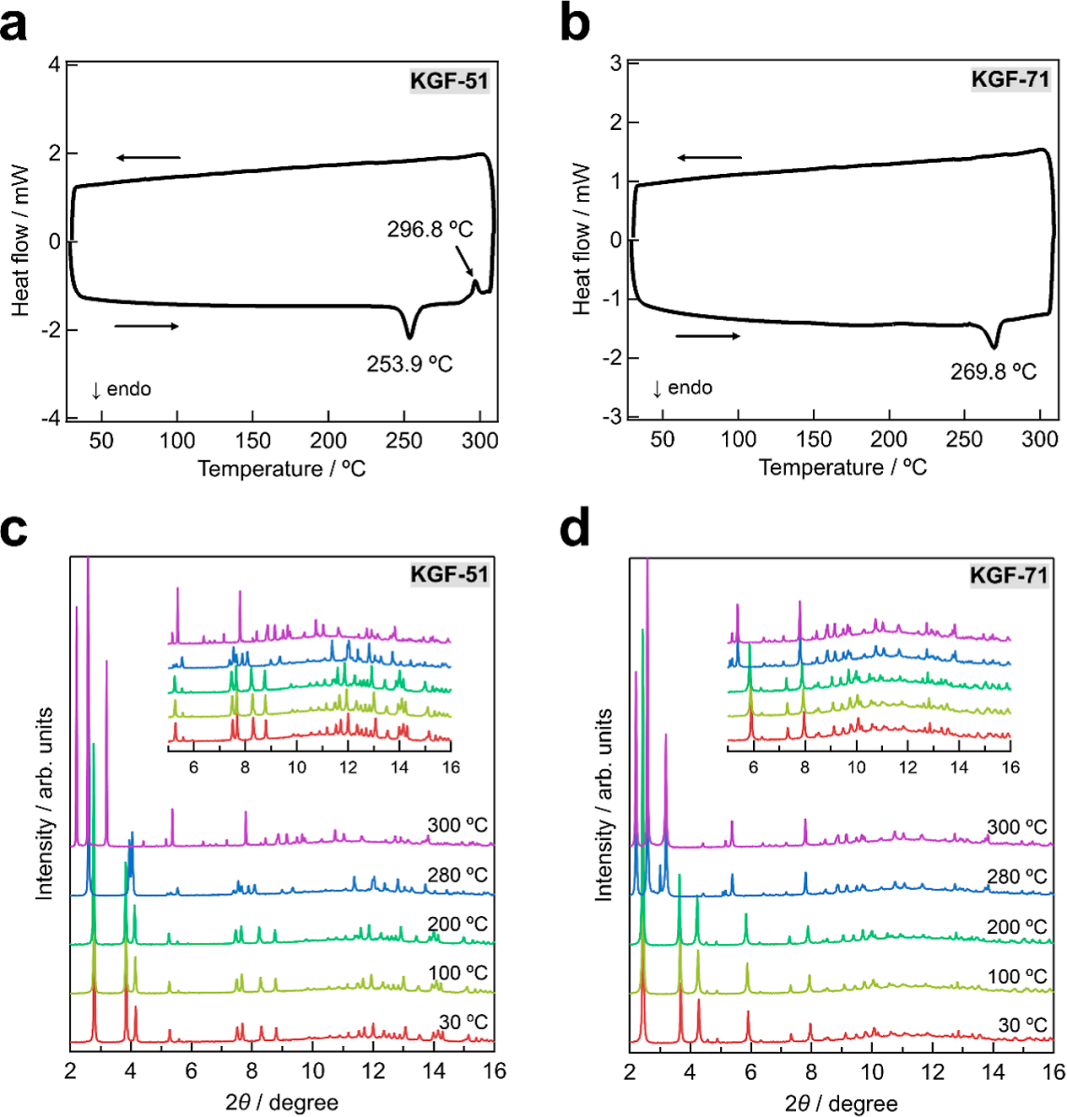
Phase transition behaviors of **KGF-51** and **KGF-71**. DSC profiles of (a) **KGF-51** and (b) **KGF-71** in the first cycle. VT-PXRD patterns of (c) **KGF-51** and (d) **KGF-71**.

Collectively, the solvothermal reaction between the Cd­(II) ion and 2-HSNPh ligand yields two polymorphs with different assembled structures. Specifically, **KGF-51** featured a single-strand ladder structure, whereas **KGF-71** featured a double-strand chain structure. The pure powders for the characterization of physical properties were obtained by optimizing the reaction conditions including synthetic solvents, reaction temperature, and time (see the Experimental Section). The purity of the obtained products was fully confirmed by elemental analysis and powder X-ray diffraction (PXRD) (Figure S5).

The thermophysical properties of **KGF-51** and **KGF-71** were investigated through thermogravimetric (TG) analysis, differential scanning calorimetry (DSC), and variable-temperature PXRD (VT-PXRD). As illustrated in Figure S6, no weight loss was observed up to 300 °C for **KGF-51** and up to 330 °C for **KGF-71**. Notably, both **KGF-51** and **KGF-71** exhibit small DSC peaks before thermal decomposition. Repeated DSC profiles were collected in the temperature range 30–310 °C. In the first heating process, the DSC curve of **KGF-51** displays endothermic and exothermic peaks at 253.9 and 296.8 °C, respectively, whereas that obtained for **KGF-71** exhibits a single endothermic peak at 269.8 °C (Figures 2a,b and S7). To further investigate the resultant phase transition behaviors, synchrotron VT-PXRD measurements were performed. As expected, the structure of **KGF-51** remained stable up to 240 °C. Upon further heating, the PXRD patterns of **KGF-51** underwent a drastic change at 280 °C and again at 300 °C (Figures 2c, S8a). In contrast, the PXRD patterns of **KGF-71** changed only once during heating (Figures 2d, S8b). Notably, the PXRD patterns after heating to 300 °C remained unchanged upon cooling back to room temperature (Figure S9). These results are fully consistent with the DSC results. Notably, VT-PXRD also demonstrated that **KGF-51** and **KGF-71** irreversibly transformed into the same product (Figure S9), with **KGF-51** undergoing an intermediate phase during the transition. The product after the irreversible structural transformation is designated as **KGF-79**.

Since the microcrystals of **KGF-79**, obtained via thermal treatment of **KGF-51** and **KGF-71**, were not suitable for SCXRD analysis (Figure S1c), its crystal structure was determined using the microcrystal electron diffraction (MicroED) method. The sample for MicroED measurement was prepared by heating pure **KGF-51** or **KGF-71** powder at 300 °C for 5 min under a N_2_ atmosphere and then cooling to room temperature. The MicroED results also confirmed that both **KGF-51** and **KGF-71** had the same structure after thermal treatment. **KGF-79** crystallized in the monoclinic *C*2 (polar space group) with the asymmetric unit containing four Cd­(II) ions and six 2-SNPh^–^ anions (Figure 3a and Table S1). Among the four 2-SNPh^–^ ligands, two exhibited disorder. Similar to **KGF-51** and **KGF-71**, the Cd centers adopted a [CdS_4_]-based tetrahedral coordination geometry (Figure S2c), with Cd–S distances of 2.46–2.65 Å. All S atoms serve as μ_2_-S bridging atoms, forming an inorganic (−Cd–S−)_*n*_ chain (Figure S3c). Interestingly, three of these 1D chains were further assembled through interchain S···S interactions, resulting in the formation of a triple-strand chain (Figure 3b,c). The S···S distances are 3.14 and 3.15 Å, which are comparable to previously reported Cd­(II) S-CPs.^29^ The central 1D chain consists of an inorganic (−Cd–S−)_*n*_ chain where Cd1 and Cd2 are alternatively arranged, while the side 1D chains comprise an inorganic (−Cd–S−)_*n*_ chain whose Cd3 and Cd4 are aligned alternatively (Figure S4c). Le Bail analysis of the PXRD patterns obtained at 300 °C revealed a unit cell consistent with that obtained from the MicroED analysis.

**Figure 3:**
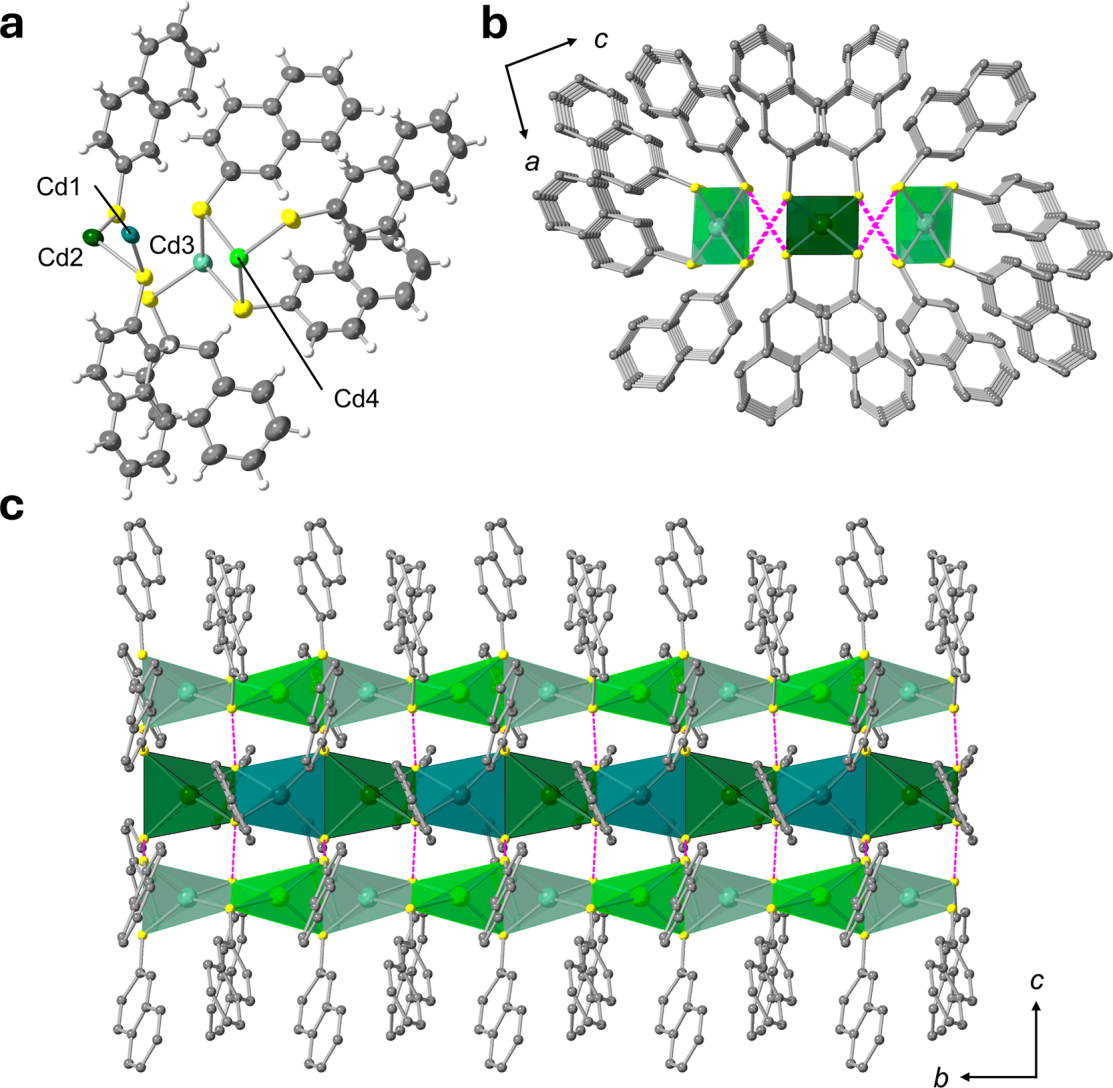
MicroED analysis of **KGF-79**. (a) Asymmetric unit and (b,c) 1D chain structure. Color code: Cd, green; S, yellow; C, gray; H, white. Purple lines indicate the S···S interactions between (−Cd–S−)_*n*_ chains. The structures are structural models generated based on the major occupancy configurations observed in the crystal structure.

Overall, both **KGF-51** with single-strand ladder chains and **KGF-71** with double-strand chains underwent a thermally induced structural transformation to **KGF-79** with triple-strand chains. In our previous study, Cd­(II) S-CPs with HSPhOMe isomers exhibited structural variations in the (−Cd–S−)_*n*_ assembly depending on the position of the methoxy substituent. Specifically, as the steric effect increased, the number of assembled (−Cd–S−)_*n*_ chains decreased; a single-strand chain formed at the ortho-position, while double- and triple-strand chains formed at the meta- and para-positions, respectively. In the present study, three polymorphs were observed, namely, a single-strand ladder, a double-strand chain, and triple-strand chains, despite using the identical ligand. Considering that the 2-HSNPh ligand has a structure in which the benzene ring is positioned between meta- and para-positions relative to the HSPh skeleton, its steric hindrance is likely intermediate between those of *m*- and *p*-HSPhOMe. This intermediate steric effect promotes the formation of both double-strand (−Cd–S−)_*n*_ and triple-strand (−Cd–S−)_*n*_ chains. Furthermore, the reaction between Cd­(II) ion and 1-HSNPh (whose benzene ring is located between ortho- and meta-positions relative to the HSPh skeleton) yielded a pure single-phase (**KGF-88**) containing a single-strand (−Cd–S−)_*n*_ chain (Figure S10). These results clearly demonstrate that moderate steric effects lead to multiple polymorphisms. As mentioned above, polymorphism in Pb­(II) S-CPs is well known, owing to the ability of the Pb­(II) center to adopt various coordination numbers and geometries.^30,31^ Conversely, polymorphism in Cd­(II)-thiolate coordination polymers is rare as Cd­(II) centers typically favor a tetrahedral coordination geometry.

Dynamic structural transformations are often accompanied by changes in the physicochemical properties of materials. In the present study, the transition from the centrosymmetric (*P*1̅) to the noncentrosymmetric space group (*C*2) appears to be associated with the emergence of nonlinear optical (NLO) properties, such as SHG. Indeed, SHG experiments revealed that both **KGF-51** and **KGF-71** exhibit no SHG activity, while **KGF-79** shows distinct SHG activity (Figure 4). The SHG-active **KGF-79** thus holds promise for application in NLO materials. Similar SHG-related structural transformations have been observed in 1D coordination polymers.^31,34^

**Figure 4:**
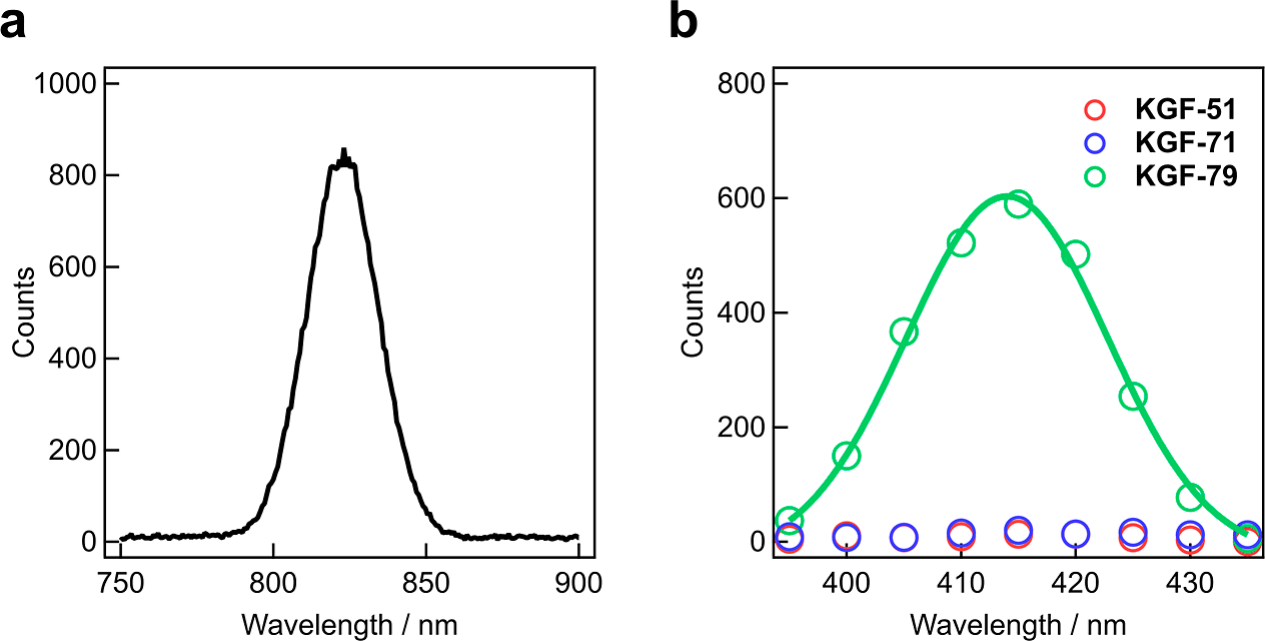
(a) Excitation pulse laser utilized for the SHG experiments. (b) SHG spectra of **KGF-51** (red), **KGF-71** (blue), and **KGF-79** (green).

The presence of an inorganic (−Cd–S−)_*n*_ chain motivated us to investigate the semiconductive properties. Initially, the optical band gap energies of **KGF-51**, **KGF-71**, and **KGF-79** were evaluated through diffuse-reflectance ultraviolet–visible (DR-UV–Vis) spectroscopy. As illustrated in Figure 5a, all Cd­(II) S-CPs exhibit a similar UV absorption behavior. The optical band gap energies were estimated by Tauc function^35^ and Gaussian fitting^36^ (Figures S11–S13 and Table S2). In this study, the values obtained from the Tauc function for an indirect semiconductor were adopted, as it is commonly used to estimate the optical band gaps of CPs containing highly covalent bonds such as those with N, S, and C coordination atoms. The optical band gap energies of **KGF-51**, **KGF-71**, and **KGF-79** were determined to be 3.25, 3.29, and 3.34 eV, respectively (Figure 5a inset). Photoelectron yield spectroscopy (PYS) was employed to determine the absolute valence band maximum (VBM) level, in which the VBM levels were −5.72 eV for **KGF-51**, −5.67 eV for **KGF-71**, and −5.46 eV for **KGF-79** below the vacuum level (Figure S14). From the DR-UV–Vis results, the absolute conduction band minimum (CBM) levels of **KGF-51**, **KGF-71**, and **KGF-79** were determined to be −2.47, −2.38, and −2.12 eV, respectively. The electronic energy levels under the vacuum level are summarized in Figure 5b. The VBM energy levels of **KGF-51**, **KGF-71**, and **KGF-79**, which incorporate 2-naphthalenethiol, are higher than those obtained for Cd­(II) S-CPs with HSPhOMe isomers.^29^ This is attributed to the presence of extended π-conjugation systems, which elevate the VBM level. Considering that the CBM of **KGF-51**, **KGF-71**, and **KGF-79** is located above the reduction potential of H^+^/H_2_, they could potentially generate hydrogen via water splitting, as reported in the literature.^37^

**Figure 5:**
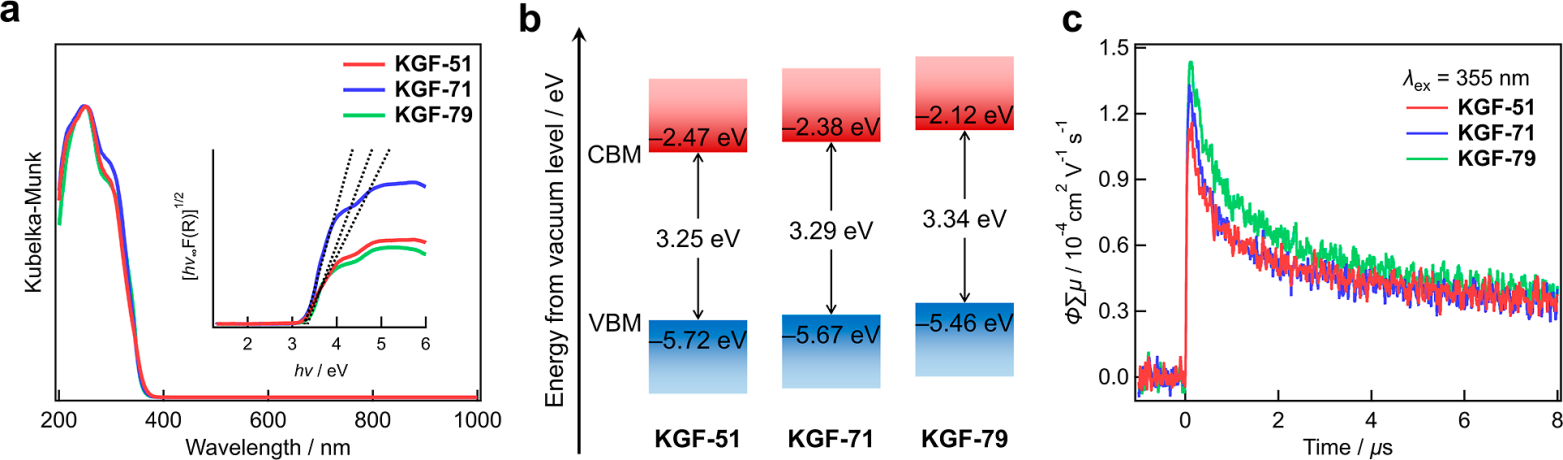
Semiconductive properties of **KGF-51**, **KGF-71**, and **KGF-79**. (a) DR-UV–Vis spectroscopy. Inset shows Tauc plots for the indirect semiconductor. (b) Energy diagram under the vacuum level. (c) TRMC results (λ_ex_ = 355 nm).

The photoconductive properties of the S-CPs were investigated through TRMC experiments. All S-CPs exhibited distinct TRMC signals with similar ΦΣμ_max_ values (Figure 5c). The ΦΣμ maximum values were 1.16 × 10^–4^ cm^2^ V^–1^ s^–1^ for **KGF-51**, 1.33 × 10^–4^ cm^2^ V^–1^ s^–1^ for **KGF-71**, and 1.44 × 10^–4^ cm^2^ V^–1^ s^–1^ for **KGF-79**. These results clearly suggest that the photoconductive nature is maintained, even after structural transformation.

To elucidate their photoconductive mechanism, we performed first-principles calculations using the CASTEP software (details are described in the Experimental Section).^38^ The simulated band structure indicated that all Cd­(II) S-CPs exhibit flat bands with a small dispersion slope (Figures S15–S18). Density of states (DOS) analysis revealed that the VBM was mainly composed of C and S atoms with a minor contribution from Cd atoms, whereas the CBM was dominated by C atoms with small contributions from Cd and S atoms (Table S3). The distributions of the VBM and CBM further support the DOS analysis, indicating that both are primarily located on the 2-SNPh^–^ ligands. Given the absence of π–π interactions between 2-SNPh^–^ ligands that behave as a conduction pathway, the observed photoconductivity is likely attributed to the charge mobility along the inorganic (−Cd–S−)_*n*_ chains via a hopping mechanism. Similar electron mobility originating from the (−Cd–S−)_*n*_ network has also been observed in Cd­(II) S-CPs based on HSPhOMe derivatives.^29^ In addition, the photoconductivity originating from inorganic (−Cd–S−)_*n*_ chains is retained even after structural transformation of nonpolar **KGF-51** and **KGF-71** into polar **KGF-79**. Photoconductive **KGF**-**79**, with its polar structure, holds potential for exhibiting the bulk photovoltaic effect.

## Conclusion

We synthesized and characterized three polymorphs of a semiconductive 1D Cd­(II) S-CP. **KGF-51** demonstrated the formation of single-strand (−Cd–S−)_*n*_ chains with a nonpolar space group, whereas **KGF-71** adopted double-strand (−Cd–S−)_*n*_ chains with a nonpolar space group. Thermophysical analysis revealed that both **KGF-51** and **KGF-71** underwent thermally induced structural transformation into **KGF-79** containing triple-strand (−Cd–S−)_*n*_ chains. Notably, this structural transformation is accompanied by the transition from a nonpolar to a polar structure. TRMC experiments and first-principles calculations revealed that **KGF-51**, **KGF-71**, and **KGF-79** exhibit photoconductivity originating from the inorganic (−Cd–S−)_*n*_ chains through a hopping mechanism. This study represents the first report on polymorphic Cd­(II) S-CP with different inorganic (−Cd–S−)_*n*_ assemblies. Therefore, our findings provide valuable insights into the design strategy for the crystal engineering of semiconductive Cd­(II) S-CPs.

## Experimental Section

### Materials

Cd­(NO_3_)_2_·4H_2_O (98%) was purchased from Sigma-Aldrich. 1-Naphthalenethiol (1-HSNPh) (>98.0%) and 2-naphthalenethiol (2-HSNPh) (>98.0%) were purchased from Tokyo Chemical Industry Co. Methanol (MeOH) (≥99.8%), ethanol (EtOH) (≥99.5%), *N*,*N*-dimethylformamide (DMF) (≥99.5%), and acetone (≥99.0%) were purchased from FUJIFILM Wako Pure Chemical Industries. All chemicals and solvents employed in the syntheses were of reagent grade and were used without further purification.

### Syntheses

#### Single Crystals of **KGF-51**

Cd­(NO_3_)_2_·4H_2_O (23.5 mg, 0.08 mmol) and 2-HSNPh (24.3 mg, 0.15 mmol) were dissolved in EtOH/DMF (2 mL; v/v = 1:1). The solution was heated at 80 °C for 48 h in a sealed autoclave and cooled to 30 °C over 12 h to yield **KGF-51** as colorless, needle-like crystals containing **KGF-71**.

#### Single Crystals of **KGF-71**

Cd­(NO_3_)_2_·4H_2_O (46.3 mg, 0.15 mmol) and 2-HSNPh (48.3 mg, 0.30 mmol) were dissolved in MeOH/DMF (2 mL; v/v = 1:1). The solution was heated at 80 °C for 48 h in a sealed autoclave and cooled to 30 °C over 12 h to yield **KGF-71** as colorless, needle-like crystals.

#### Single Crystals of **KGF-88**

CdI_2_ (54.9 mg, 0.15 mmol) and 1-HSNPh (48.1 mg, 0.30 mmol) were dissolved in H_2_O (2 mL). The solution was heated at 80 °C for 48 h in a sealed autoclave and cooled to 30 °C over 12 h to yield **KGF-88** as colorless, needle-like crystals.

#### Pure **KGF-51** Powder

A solution of 2-HSNPh (48.1 mg, 0.30 mmol) in DMF (2 mL) was added to a solution of Cd­(NO_3_)_2_·4H_2_O (46.4 mg, 0.15 mmol) in MeOH (2 mL) at room temperature, and the reaction mixture was stirred for 24 h at 60 °C under ambient pressure. The resulting white solid was collected by centrifugation (4000 rpm, 3 min), washed with acetone, and dried under a vacuum to obtain pure **KGF-51** as a white powder. Yield: 52.2 mg (80.5%). Anal. C_20_H_14_CdS_2_: calcd C, 55.75; H, 3.28; N, 0.00. Found: C, 55.51; H, 3.27; N, 0.00.

#### Pure **KGF-71** Powder

A solution of Cd­(NO_3_)_2_·4H_2_O (61.6 mg, 0.20 mmol) in MeOH (3.8 mL) was added to a solution of 2-HSNPh (64.1 mg, 0.40 mmol) in DMF (0.2 mL) at room temperature in an autoclave. The solution was heated for 48 h at 80 °C in a sealed autoclave and then cooled to 30 °C over 12 h. The resulting solid was collected by centrifugation (4000 rpm, 3 min), washed with acetone until the yellow solid disappeared, and dried under vacuum to obtain pure **KGF-71** as a white powder. Yield: 24.8 mg (28.7%). Anal. C_20_H_14_CdS_2_: calcd C, 55.75; H, 3.28; N, 0.00. Found: C, 55.59; H, 3.34; N, 0.00.

#### Pure **KGF-79** Powder

Pure **KGF-71** powder was heated at 300 °C for 5 min in a N_2_ atmosphere to obtain pure **KGF-79** as a white powder suitable for MicroED analysis. Yield: 100%. Anal. C_20_H_14_CdS_2_: calcd C, 55.75; H, 3.28; N, 0.00. Found: C, 55.43; H, 3.28; N, 0.00.

## Methods

### Elemental Analysis

Elemental analysis was carried out at A-Rabbit-Science Japan Co., Ltd.

### Single-Crystal X-ray Diffraction (SCXRD)

SCXRD data were collected on a Rigaku Saturn CCD diffractometer by using Mo *K*α radiation (λ = 0.71075 Å). The diffraction profiles were integrated using the CrysAlisPro software. Crystal structures were solved via direct methods using the SHELXT program and refined with SHELXL.^39,40^ Anisotropic thermal parameters were used to refine all of the non-H atoms. All calculations were conducted using the Olex2 crystallographic software package.^41^

### Synchrotron PXRD (PXRD)

PXRD patterns were collected using a powder diffractometer equipped with MYTHEN detectors installed at the BL02B2 beamline of SPring-8, Japan.^42^ Powder samples were packed into borosilicate glass capillaries with an outer diameter of 0.3 mm for **KGF-51** and 0.5 mm for **KGF-71** and a glass wall thickness of 0.01 mm. The wavelength of an incident X-ray was determined to be 0.799997 Å using a standard material (CeO_2_). The sample temperature was controlled by using a N_2_ gas blower. The VT-PXRD patterns were obtained in the temperature range 30–300 °C. The heating rates were 10 °C min^–1^. Upon reaching the target temperature, the sample temperature was held constant for 60 s for temperature stabilization. The exposure time for PXRD measurements was 30 s for each measurement.

### Microcrystalline Electron Diffraction (MicroED)

**KGF-79** powder, prepared from **KGF-51** or **KGF-71**, was dusted on QuantiFoil Mo R1.2/1.3 grids and loaded to a Talos Arctica microscope cooled to ∼79 K. **KGF-79** particles comprised aggregated bundles of thin plates (Figure S19). The microscope was operated at 200 kV and controlled by SerialEM.^43^ Diffraction images were recorded on a Falcon3 direct electron detector in the linear mode,^44^ while the stage was rotated at 0.435° rotation per fraction (∼0.95°/s). The electron flux was ∼0.06 electron/Å^2^/s. The virtual detector distance was ∼615.5 mm. Diffraction patterns were indexed and integrated with DIALS.^45,46^ GNU parallel^47^ was utilized for parallel processing. **KGF-79** prepared from **KGF-51** (two grids) and **KGF-71** (one grid) gave the same unit cell constants (Figure S20). Of the 473 particles measured on the two grids of **KGF-79** prepared from **KGF-51**, 355 crystals were indexed consistently, and 63 high-quality, highly isomorphic crystals were selected by xia2.multiplex,^48^ scaled with dials.scale,^49^ and merged (crystallographic merging statistics are provided in Table S4). Structures were phased by SHELXT^39^ and kinematically refined by olex2 using the Olex2 GUI.^41^ Anisotropic thermal parameters were used to refine all non-H atoms. The naphthalene ring was treated with AFIX 116 constraints and SADI, SIMU, and RIGU restraints. Hydrogen atoms were placed in idealized positions and refined by using a riding model.

### Scanning Electron Microscopy (SEM)

Scanning electron microscopy images were acquired on a JEOL JCM-6000 system. All samples were sputter-coated with Au before analysis.

### Thermogravimetry–Differential Scanning Calorimetry (TG–DSC)

TG–DSC was performed on a HITACHI STA200RV instrument in the temperature range 30–600 °C at a heating rate of 10 °C min^–1^ under N_2_.

### Differential Scanning Calorimetry (DSC)

DSC was performed on a SII nanotechnology DSC 7020 instrument in the temperature range 30–310 °C at a heating rate of 10 °C min^–1^ under N_2_.

### Second Harmonic Generation (SHG) Experiment

SHG signals were measured by using a time-correlated single-photon counting system. Samples were excited by femtosecond laser pulses generated by a cavity-dumped Ti:Sapphire laser (Kapteyn-Murnane, Cascade), and the SHG signals were detected using a monochromator–microchannel plate photomultiplier setup (JASCO, CT-10, and Hamamatsu, R2809U). CF650 and CF680 filters (Asahi Spectra) were employed to minimize the effects of the scattering.

### Diffuse-Reflectance Ultraviolet–Visible (DR-UV–Vis) Spectroscopy

DR-UV–Vis spectra (200–800 nm) were recorded on a Shimadzu UV-3600 UV–Vis–NIR spectrophotometer using BaSO_4_ powder as a nonadsorbing background. The band gaps were calculated through the Kubelka–Munk (KM) function using the following equation:

$$\frac{K}{S}=F\left(R\right)=\frac{{\left(1-R\right)}^{2}}{2R}$$

where *K* is the absorption coefficient, *S* is a scattering factor, *R* is the reflectance, and *F*(*R*) is the KM function. The band gap was determined from the Tauc plot with [*F*(*R*) × *hv*]^1/2^ vs *h*ν or [*F*(*R*) × *h*ν]^2^ vs *h*ν by extrapolating the linear region to the abscissa. Gaussian fitting was performed using Igor Pro Version 9.0.5.1 software, along with the Multipeak Fitting package and a LogCubic baseline.

### Photoelectron Yield Spectroscopy (PYS)

The crystalline samples were placed on conductive carbon tape on a glass substrate. The carbon tape was connected to an earth wire in a vacuum chamber (10^–4^ Pa) of a Bunko Keiki BIP-KV202GD instrument. Monochromated ultraviolet (UV) light (4–7 eV corresponding to 350 to 177 nm) was exposed to the sample, and the photoelectrons emitted from the sample were detected. The photon number (power) of the UV light was measured by using a photodetector before the experiment.

### Time-Resolved Microwave Conductivity (TRMC)

Crystalline samples on adhesive tape were attached to a quartz substrate and positioned in the resonant cavity, where they were exposed to continuous microwave radiation at approximately 9.1 GHz. The third harmonic generation (355 nm) of a Nd:YAG laser (Continuum Inc., Surelite II, pulse duration = 5–8 ns, 10 Hz) was used as the excitation source (incident photon density *I*_0_ = 9.1 × 10^15^ photons cm^–2^ pulse^–1^). The photoconductivity (Δσ = *A* ^–1^ Δ*P*_r_ *P*_r_^–1^, where *A* is the sensitivity factor, *P*_r_, reflected microwave power, and Δ*P*_r_, change in *P*_r_ upon exposure to light) was converted into the product of the quantum yield (φ) and sum of the charge carrier mobilities Σμ (= μ_+_ + μ_–_) using the relationship φΣμ = Δσ­(*eI*_0_ *F*_light_)^−1^, where *e* and *F*_light_ are the electron charge and correction (or filling) factor, respectively. The experiments were performed at room temperature in air.

### First-Principles Calculations

First-principles calculations were performed using a CASTEP 2020 (20.1.0.5).^38^ Band structures and densities of states were calculated using the Perdew–Burke–Ernzerhof (PBE) generalized gradient approximation (GGA) functional for the exchange–correlation potential.^50^ Atomic positions were relaxed to allow for potential energy minimization. The pseudopotential plane-wave method used an ultrasoft state in the PBE functional. The plane-wave basis set cutoff was 450.0 eV for **KGF-51**, 450.0 eV for **KGF-51**, and 326.5 eV for **KGF-79**. The Brillouin zone was sampled with a 13 × 4 × 3 *k*-point mesh for **KGF-51**, 5 × 3 × 2 *k*-point mesh for **KGF-71**, and 2 × 2 × 1 *k*-point mesh for **KGF-79**. For **KGF-79**, a structural model was constructed by taking into account the disorder of the ligands, and calculations were performed.

## Supplementary Material



## Notes

SEM textures; crystallographic data; PXRD patterns of bulk powders; TG–DSC profiles; repeated DSC curves; PXRD patterns of bulk powders; VT-PXRD; PXRD patterns of the samples after heating to 300 °C; optical band gap energy; PYS results; first-principles calculations; SerialEM montage images; scatter plots of unit cell constants; and crystallographic merging statistics (PDF)

## Abbreviations

KGF: Kwansei Gakuin Framework
HNPh: naphthalenethiol
