# Supplementary material for: Polymorphism and Thermally Induced Structural Transformation in Semiconducting Cd(II) Coordination Polymers
Source: Inorg Chem. 2025 Sep 2;64(36):18376–84. doi: 10.1021/acs.inorgchem.5c02821 (PMC12442071; doi:10.1021/acs.inorgchem.5c02821)
Supplement: Supplementary file 1 [file ic5c02821_si_001.pdf]

Supporting Information

Polymorphism and Thermally Induced Structural Transformation in Semiconducting Cd(II) Coordination Polymers

Ryohei Akiyoshi,*[a] Honoka Motokawa,[a†] Asuka Nishibe,[a†] Atsuki Yanai,[a] Takanori Nakane,[b,c] Akihiro Kawamoto,[b,c] Genji Kurisu,[b,c] Akinori Saeki,[d] Daichi Eguchi,[a] Naoto Tamai,[a] Yuki Mori,[e] Shogo Kawaguchi,[e] Kazuyoshi Ogasawara,[a] and Daisuke Tanaka*[a]

[a] Department of Chemistry, School of Science, Kwansei Gakuin University, 1 Gakuen Uegahara, Sanda, Hyogo 669-1330, Japan

[b] Institute for Protein Research, The University of Osaka, 3-2 Yamadaoka, Suita, Osaka 565-0871, Japan

[c] JEOL YOKOGUSHI Research Alliance Laboratories, Graduate School of Frontier Biosciences, The University of Osaka, 1-3 Yamadaoka, Suita, Osaka 565-0871, Japan

[d] Department of Applied Chemistry, Graduate School of Engineering, The University of Osaka, 2-1 Yamadaoka, Suita, Osaka 565-0871, Japan

[e] Japan Synchrotron Radiation Research Institute (JASRI), 1-1-1 Kouto, Sayo-cho, Sayo-gun, Hyogo 679-5198, Japan

† H. Motokawa and A. Nishibe contributed equally to this work.

Email: r.akiyoshi@kwansei.ac.jp (R. Akiyoshi), dtanaka@kwansei.ac.jp (D. Tanaka)

Table of Contents

Figure S1. SEM textures S3

Table S1. Crystallographic data........ S4

Figure S2. Local structure around Cd(II) ions........ S5

Figure S3. Inorganic $(-Cd-S-)_n$ chain structure S6

Figure S4. Packing structure S7

Figure S5. PXRD patterns of bulk powders........ S8

Figure S6. TG–DSC S8

Figure S7. Repeated DSC curves S9

Figure S8. VT-PXRD patterns S9

Figure S9. PXRD patterns of the samples after heating to 300 °C........ S10

Figure S10. Crystal structure of **KGF-88**........ S10

Figure S11. Optical band gap energy of **KGF-51**........ S11

Figure S12. Optical band gap energy of **KGF-71**........ S12

Figure S13. Optical band gap energy of **KGF-79**........ S13

Table S2. Summary of optical band gap energy........ S13

Figure S14. PYS spectroscopy S14

Figure S15. First-principles calculation of **KGF-51**........ S14

Figure S16. First-principles calculation of **KGF-71**........ S15

Figure S17. First-principles calculation of **KGF-79**........ S16

Figure S18. First-principles calculation of **KGF-79** reflecting the disordering S17

Table S3. PDOS ratio S18

Figure S19. SerialEM montage images of **KGF-79** S18

Figure S20. Scatter plots of unit cell constants of **KGF-79** S19

Table S4. Crystallographic merging statistics of **KGF-79** S20

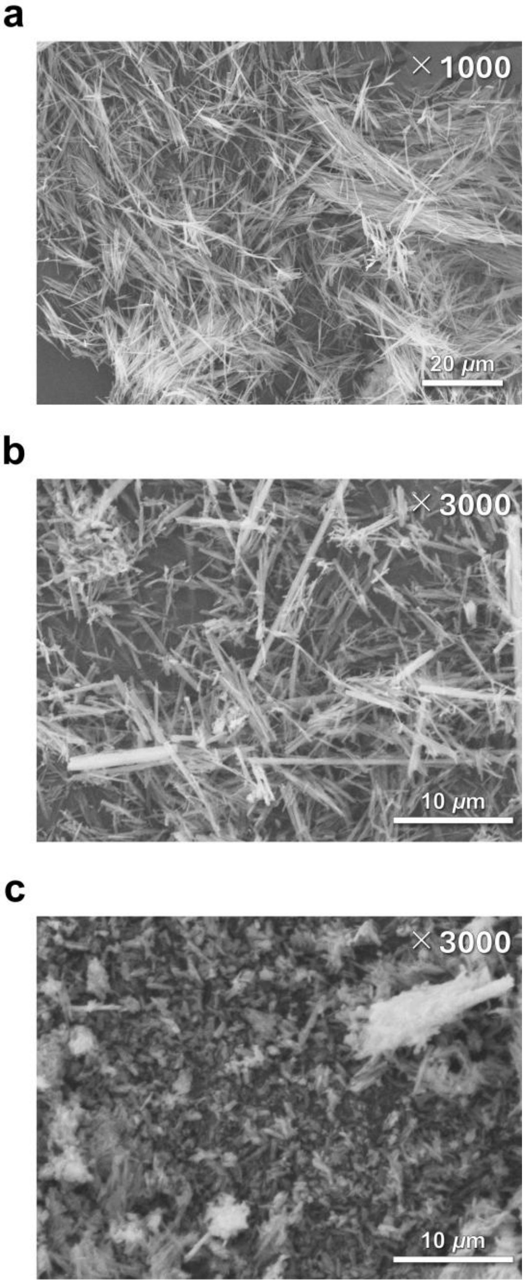

Figure S1. SEM texture of (a) **KGF-51**, (b) **KGF-71**, and (c) **KGF-79**.

Table S1. Crystallographic data for **KGF-51**, **KGF-71**, **KGF-79**, and **KGF-88**.

Compound	**KGF-51** (SCXRD)	**KGF-71** (SCXRD)	**KGF-79** (MicroED)	**KGF-88** (SCXRD)
Formula	$C_{20}H_{14}CdS_2$	$C_{20}H_{14}CdS_2$	$C_{20}H_{14}CdS_2$	$C_{20}H_{14}CdS_2$
Formula weight	430.83	430.83	430.87	430.83
T / K	150	150	79	150
Crystal system	Triclinic	Triclinic	Monoclinic	Tetragonal
Space group	$P\bar{1}$	$P\bar{1}$	$C2$	$I\bar{4}$
a / Å	3.95780(10)	7.1216(3)	35.2580(13)	15.4685(4)
b / Å	12.2608(4)	12.2667(5)	7.0785(2)	15.4685(4)
c / Å	16.8679(4)	18.7363(9)	19.8923(11)	6.8007(3)
α / °	104.124(2)	93.588(4)	90	90
β / °	92.744(2)	96.080(4)	94.163(5)	90
γ / °	92.489(2)	92.955(4)	90	90
V / Å^3	791.59(4)	1621.58(12)	4951.5(4)	1627.23(11)
Z	2	4	12	4
R_1 ($I > 2\sigma(I)$)	0.0299	0.0335	0.1649	0.0374
R_1 (all data)	0.0371	0.0478	0.1771	0.0553
wR_2 ($I > 2\sigma(I)$)	0.0627	0.0733	0.4356	0.0738
wR_2 (all data)	0.0657	0.0794	0.4403	0.0788
GOF	1.027	1.045	1.712	1.053

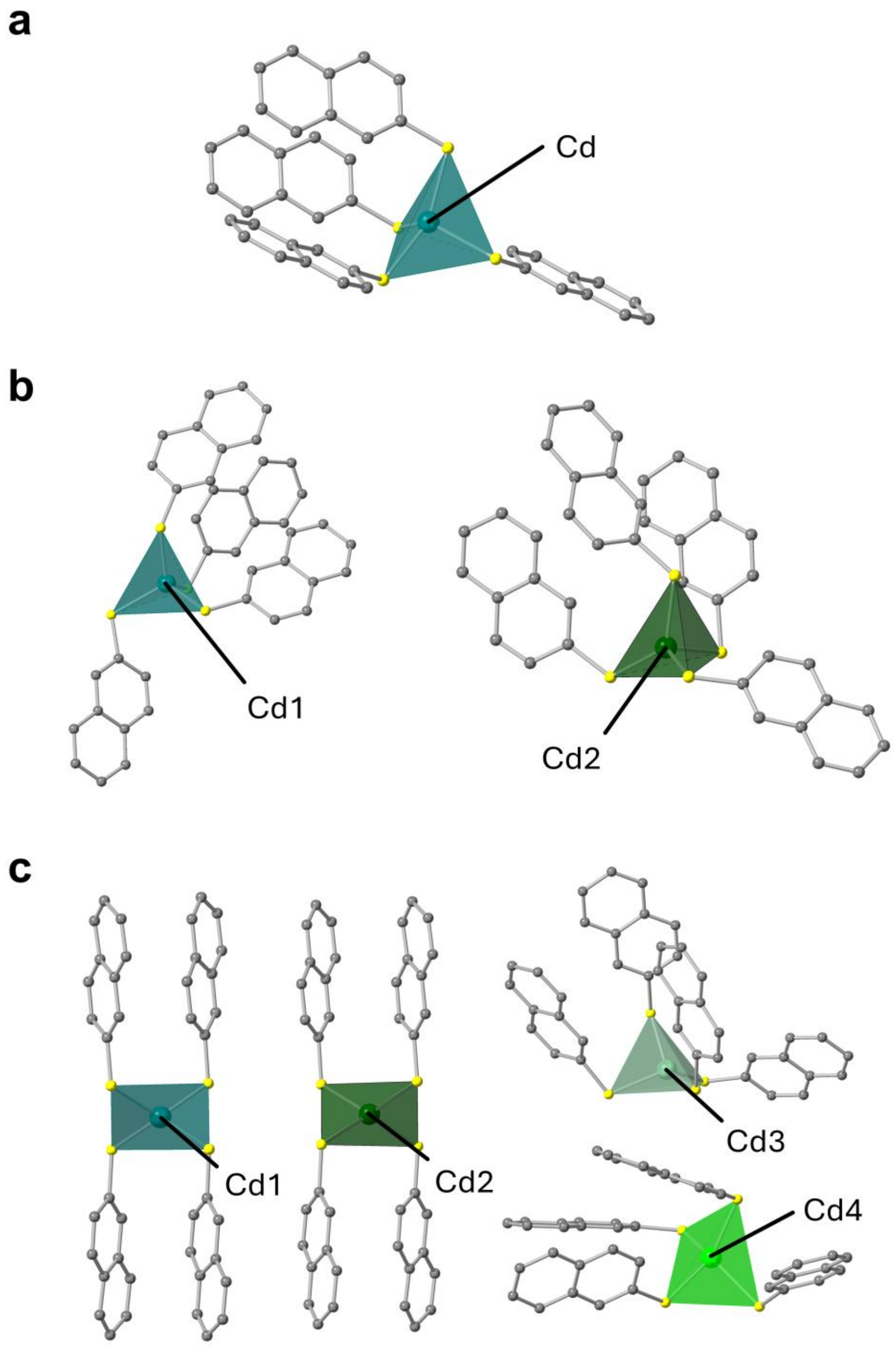

Figure S2. Local structure around Cd(II) ions in (a) **KGF-51**, (b) **KGF-71**, and (c) **KGF-79**.

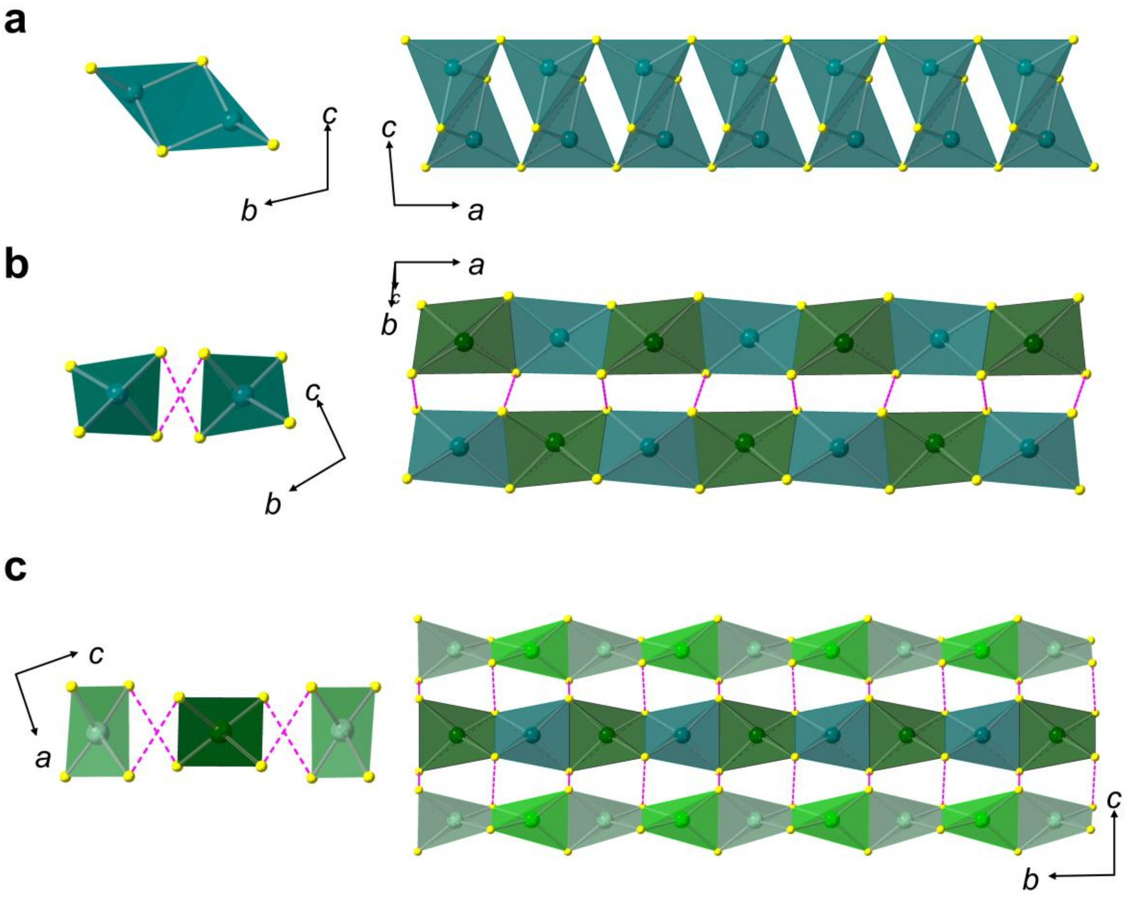

Figure S3. Structures focusing on the (–Cd–S–)$_n$ networks of (a) **KGF-51**, (b) **KGF-71**, and (c) **KGF-79**.

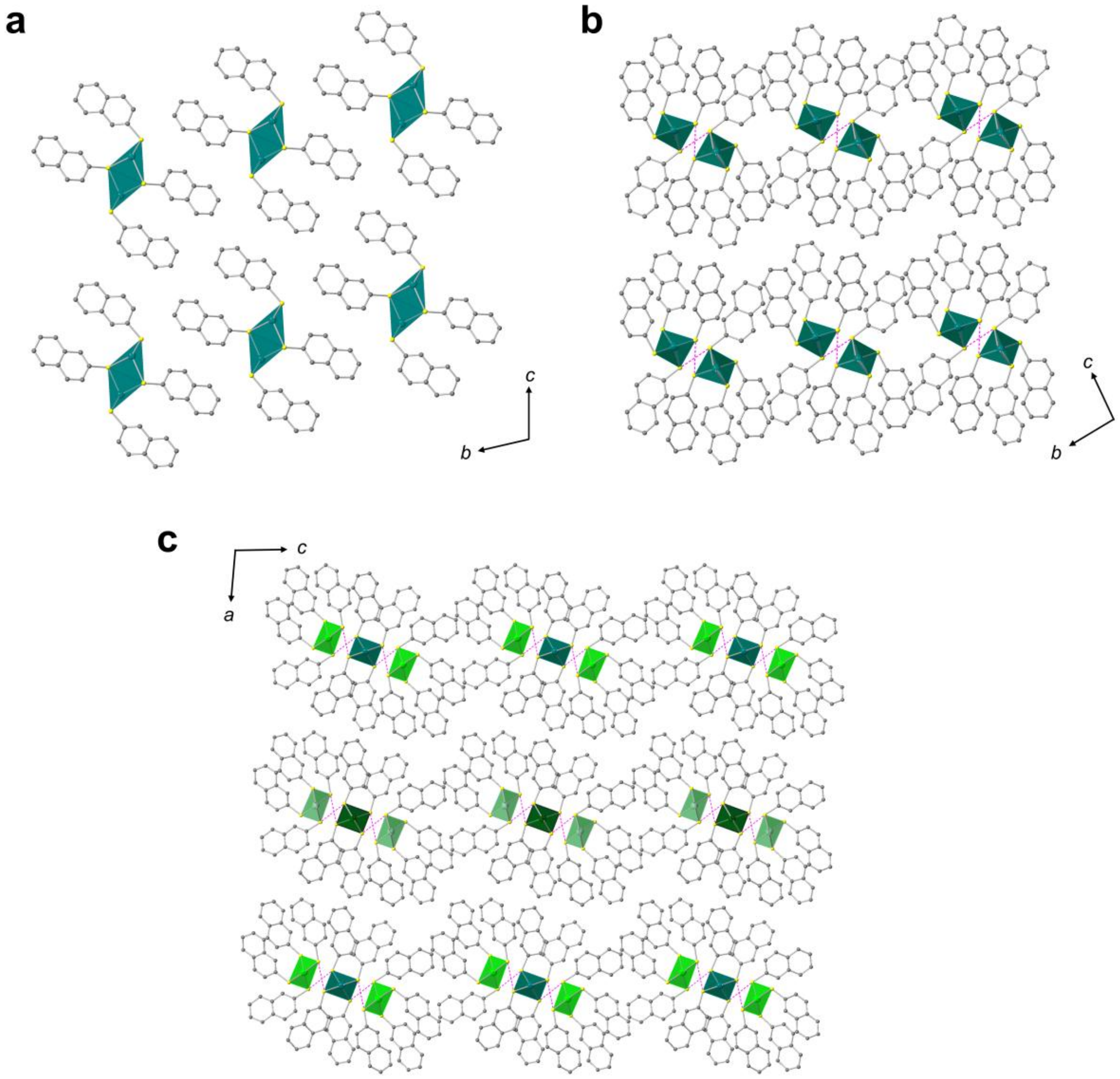

Figure S4. Packing structures of (a) **KGF-51**, (b) **KGF-71**, and (c) **KGF-79**. Purple lines indicate interchain S···S interactions.

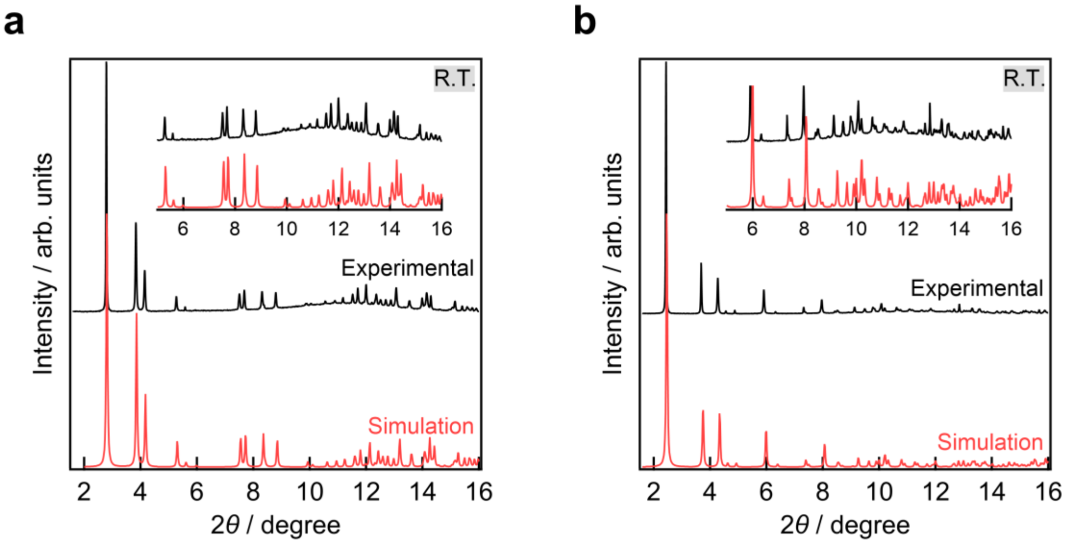

Figure S5. Room-temperature PXRD patterns of (a) **KGF-51** and (b) **KGF-71** prepared by bulk synthesis (black: experimental, red: simulation obtained from SCXRD).

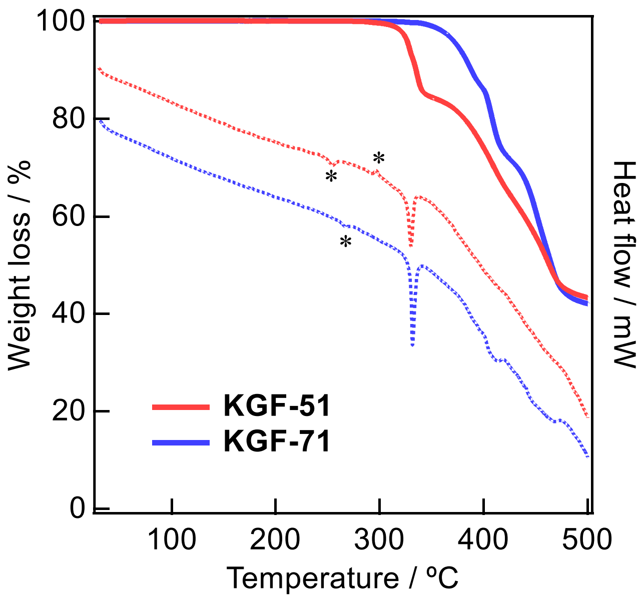

Figure S6. TG–DSC plots of **KGF-51** (red) and **KGF-71** (blue) (Solid line: TG, Dashed line: DSC). The asterisk (*) indicates the DSC peaks assignable to structural transformation.

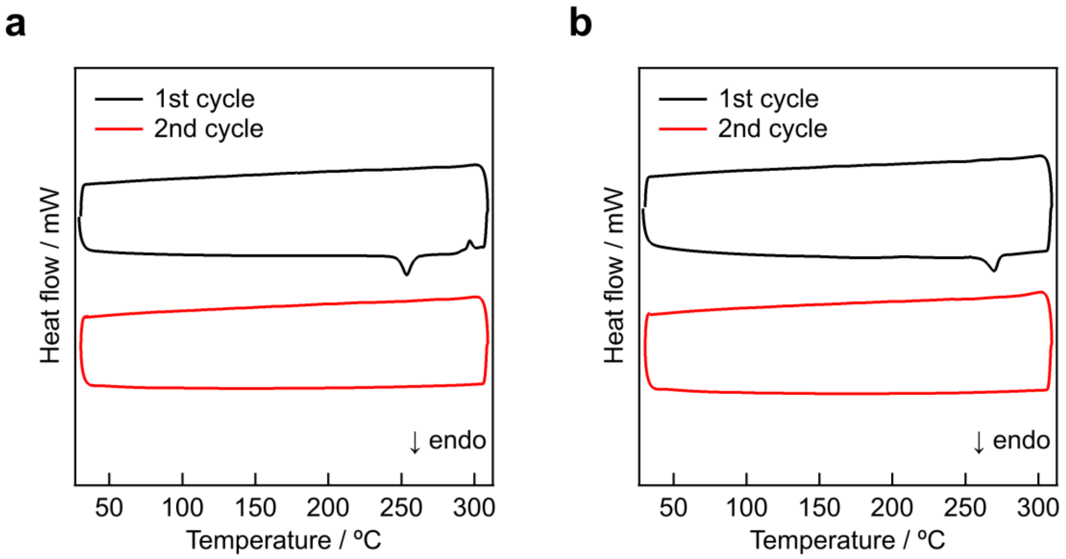

Figure S7. Repeated DSC curves of (a) **KGF-51** and (b) **KGF-71** (black: 1st cycle, red: 2nd cycle).

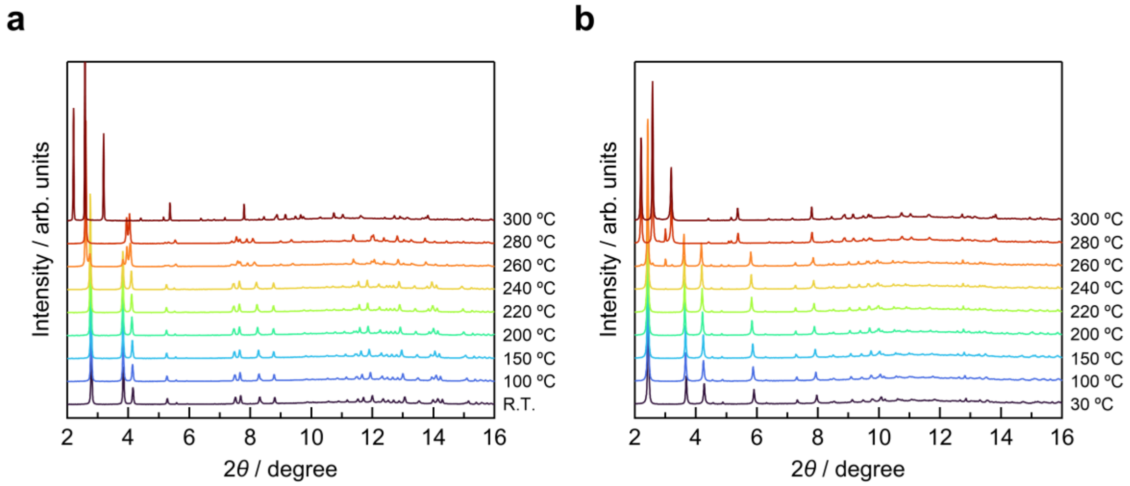

Figure S8. VT-PXRD patterns of (a) **KGF-51** and (b) **KGF-71**.

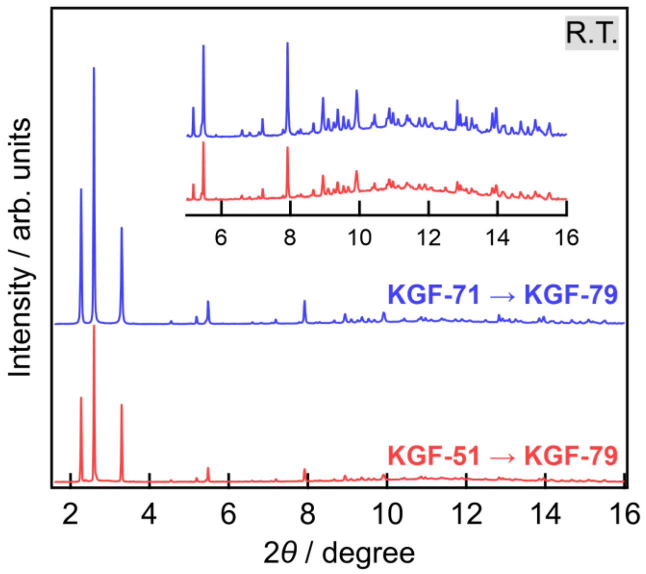

Figure S9. Room-temperature PXRD patterns of (a) **KGF-51** and (b) **KGF-71** after heating to 300 °C followed by cooling to room temperature.

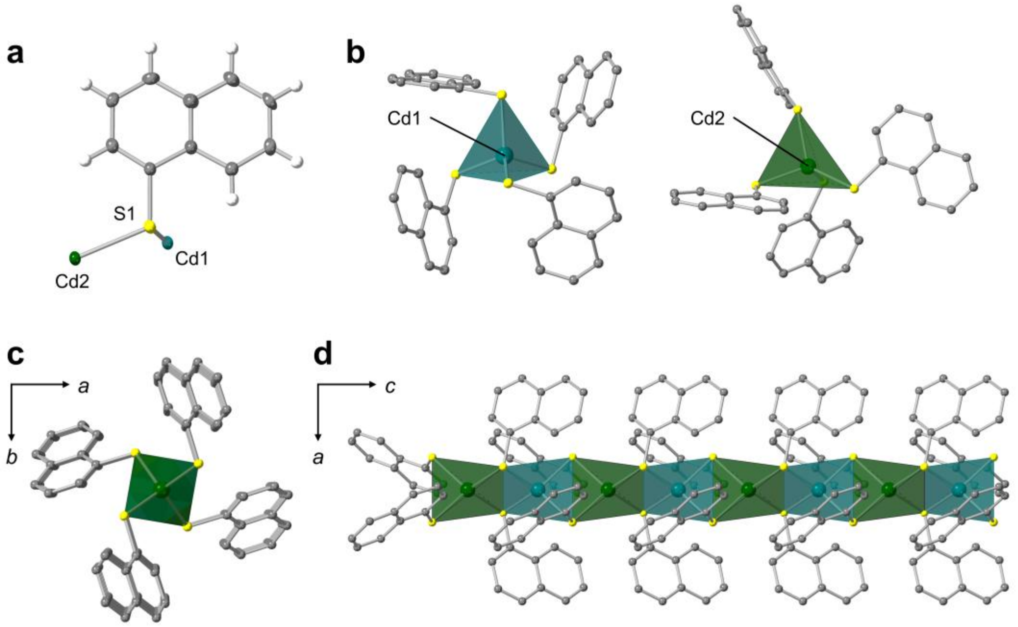

Figure S10. Crystal structure of $[Cd(1\text{-}SNPh)_2]_n$ (**KGF-88**). (a) Asymmetric unit. (b) Local structure around Cd(II) ions. (c, d) 1D chain structure.

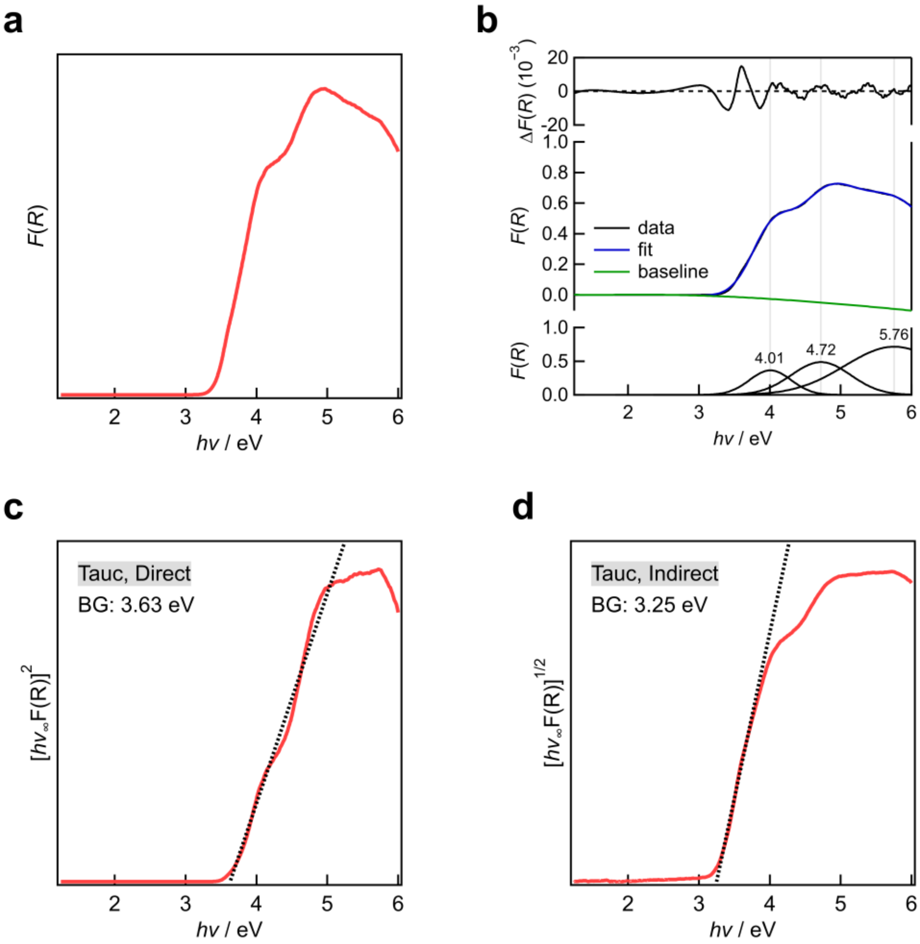

Figure. S11. Optical band gap energy of **KGF-51**. (a) DR-UV–Vis data in Kubelka–Munk units. (b) Gaussian fitting. (c) Tauc transformation and estimated indirect band gap energy. (d) Tauc transformation and estimated direct band gap.

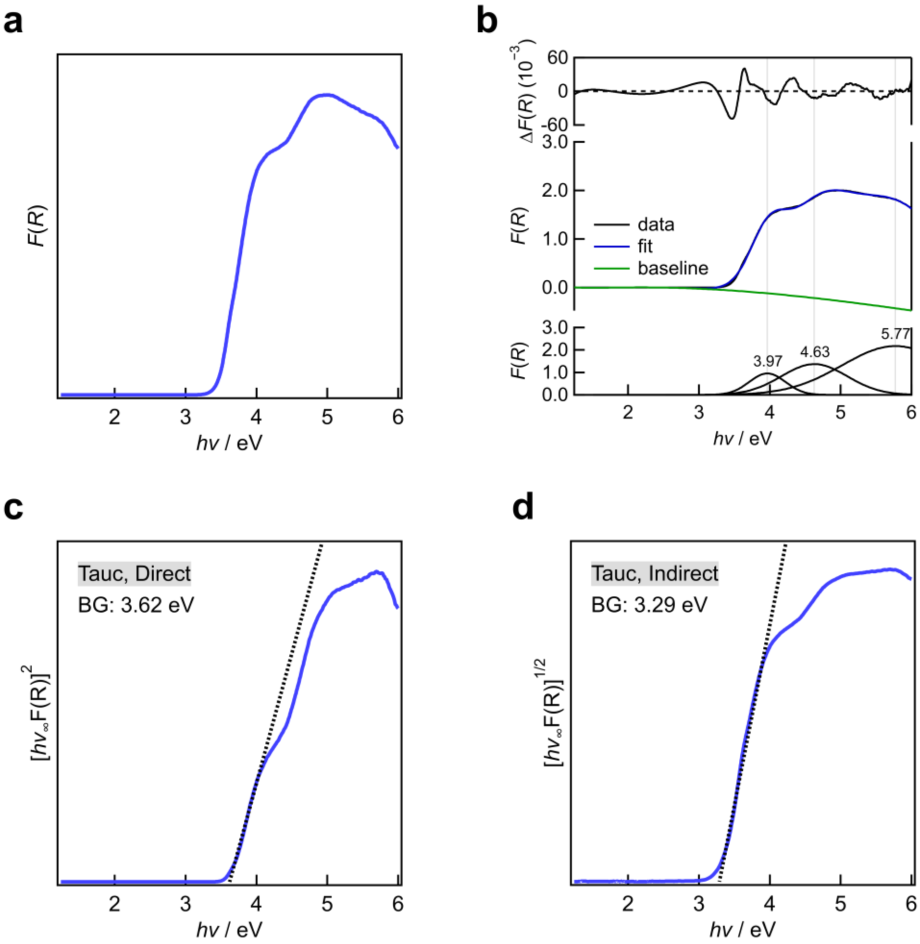

Figure. S12. Optical band gap energy of **KGF-71**. (a) DR-UV–Vis data in Kubelka–Munk units. (b) Gaussian fitting. (c) Tauc transformation and estimated indirect band gap energy. (d) Tauc transformation and estimated direct band gap.

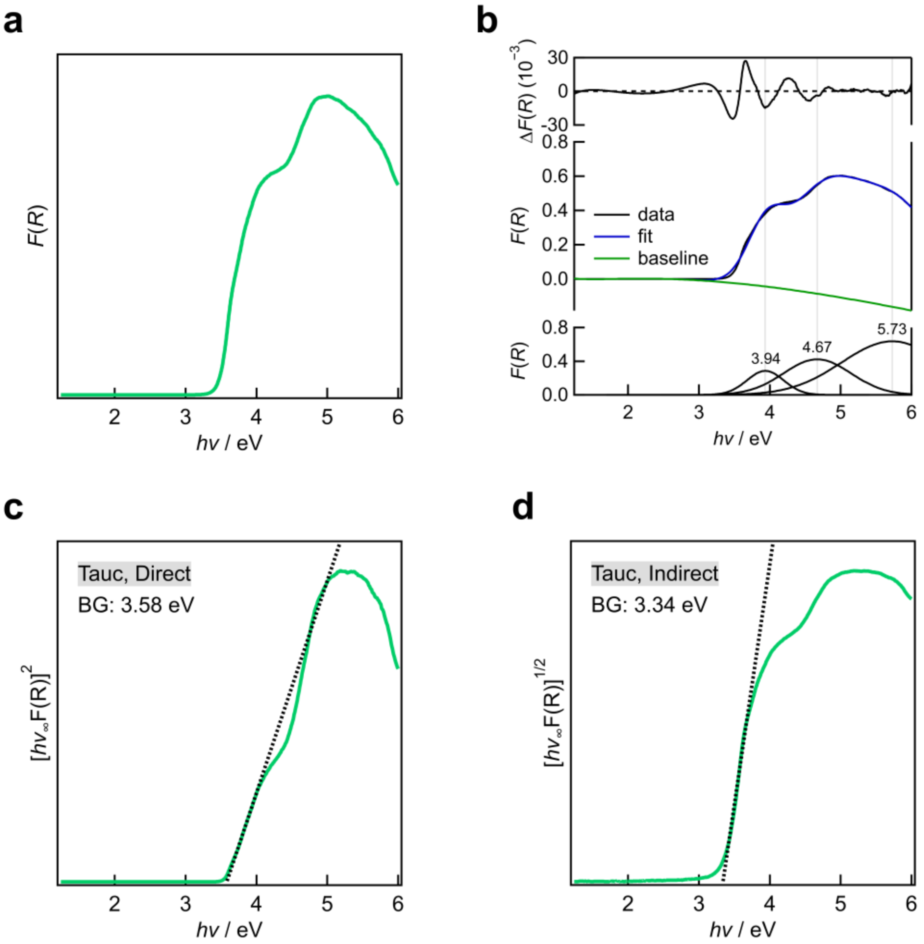

Figure. S13. Optical band gap energy of **KGF-79**. (a) DR-UV–Vis data in Kubelka–Munk units. (b) Gaussian fitting. (c) Tauc transformation and estimated indirect band gap energy. (d) Tauc transformation and estimated direct band gap.

Table S2. Summary of optical band gap energy estimated using Tauc function and gaussian fitting.

	**KGF-51**	**KGF-71**	**KGF-79**
Gaussian fitting	4.01	3.97	3.94
Tauc (Direct)	3.63	3.62	3.58
Tauc (Indirect)	3.25	3.29	3.34

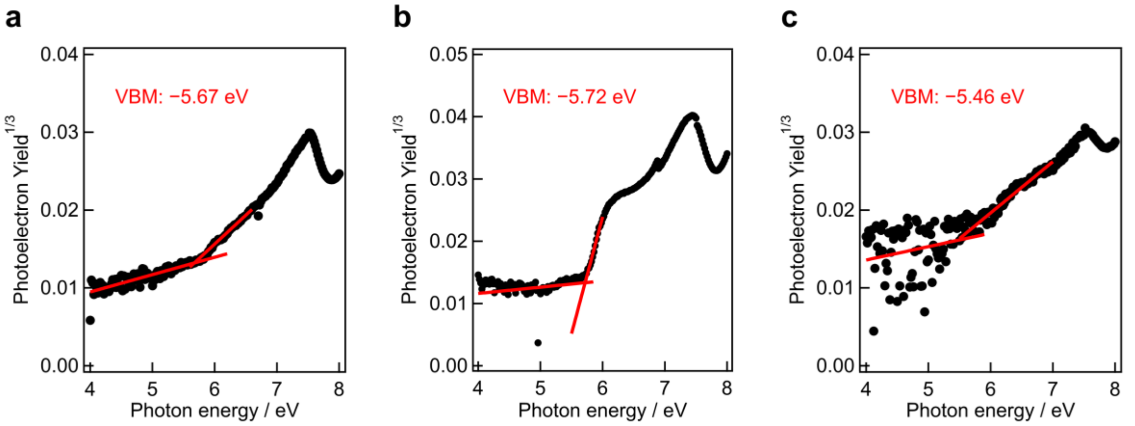

Figure S14. PYS results of (a) **KGF-51**, (b) **KGF-71**, and **KGF-79**.

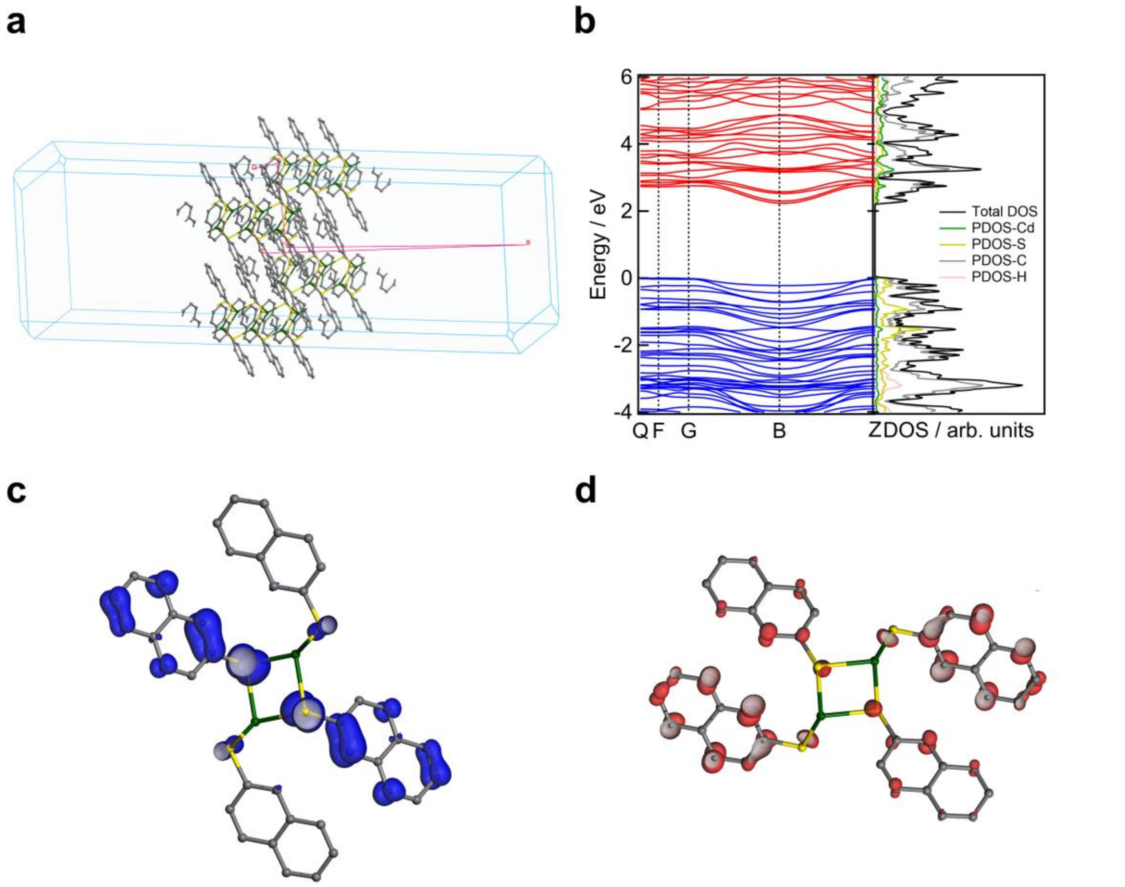

Figure S15. First-principles calculation of **KGF-51**. (a) Reciprocal space. (b) Band structure and DOS analysis. Distribution mapping of (c) VBM and (d) CBM.

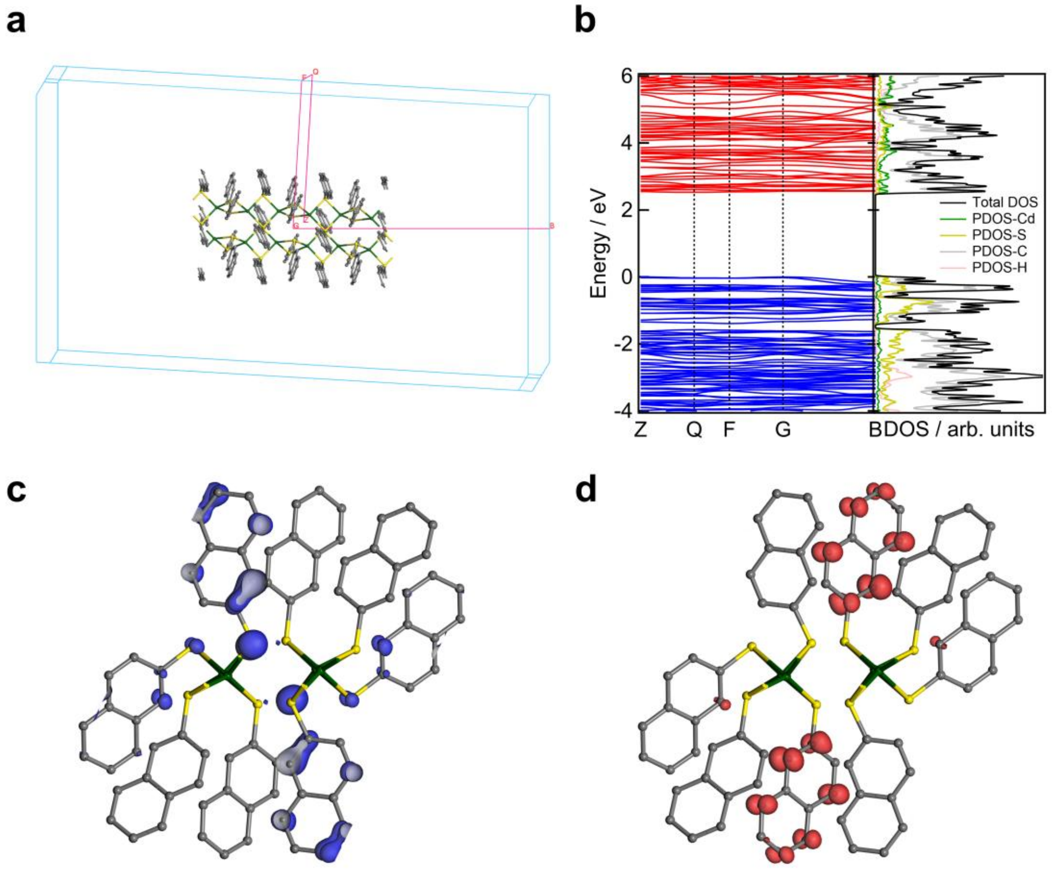

Figure S16. First-principles calculation of **KGF-71**. (a) Reciprocal space. (b) Band structure and DOS analysis. Distribution mapping of (c) VBM and (d) CBM.

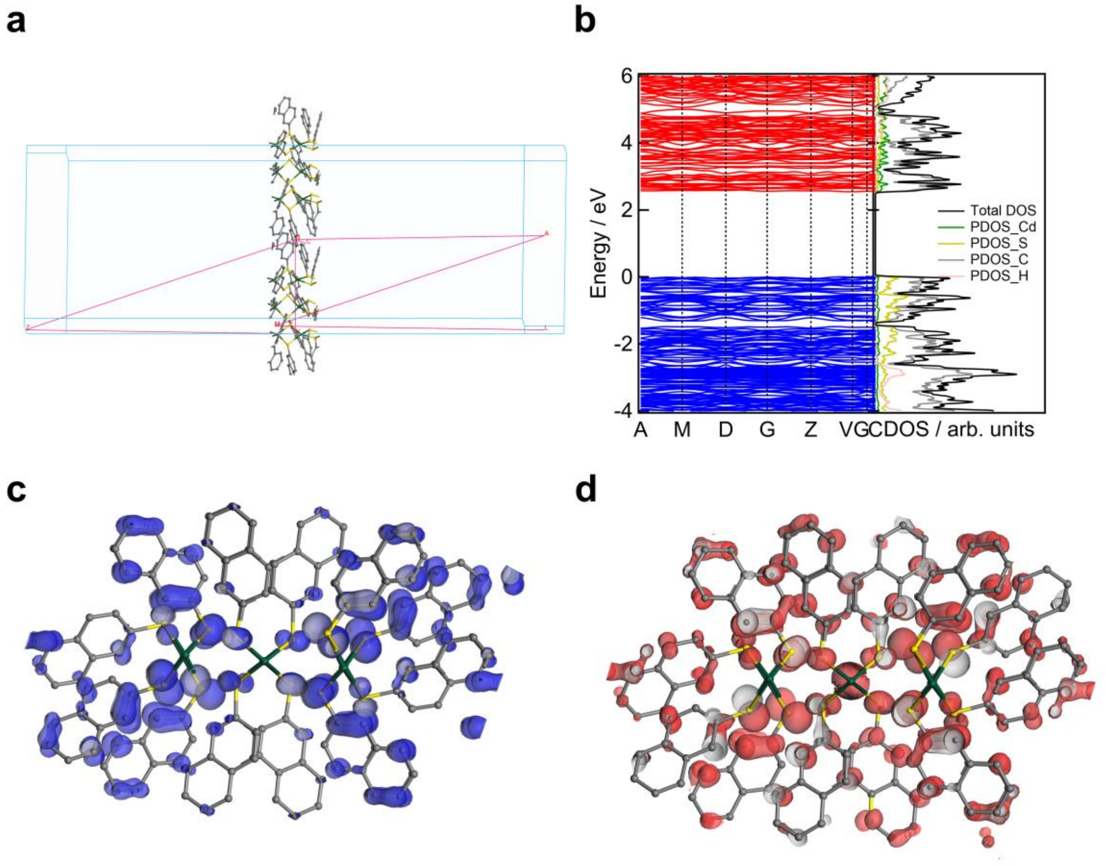

Figure S17. First-principles calculation of **KGF-79** calculated using structural models generated for major occupancy configuration observed in the crystal structure. (a) Reciprocal space. (b) Band structure and DOS analysis. Distribution mapping of (c) VBM and (d) CBM.

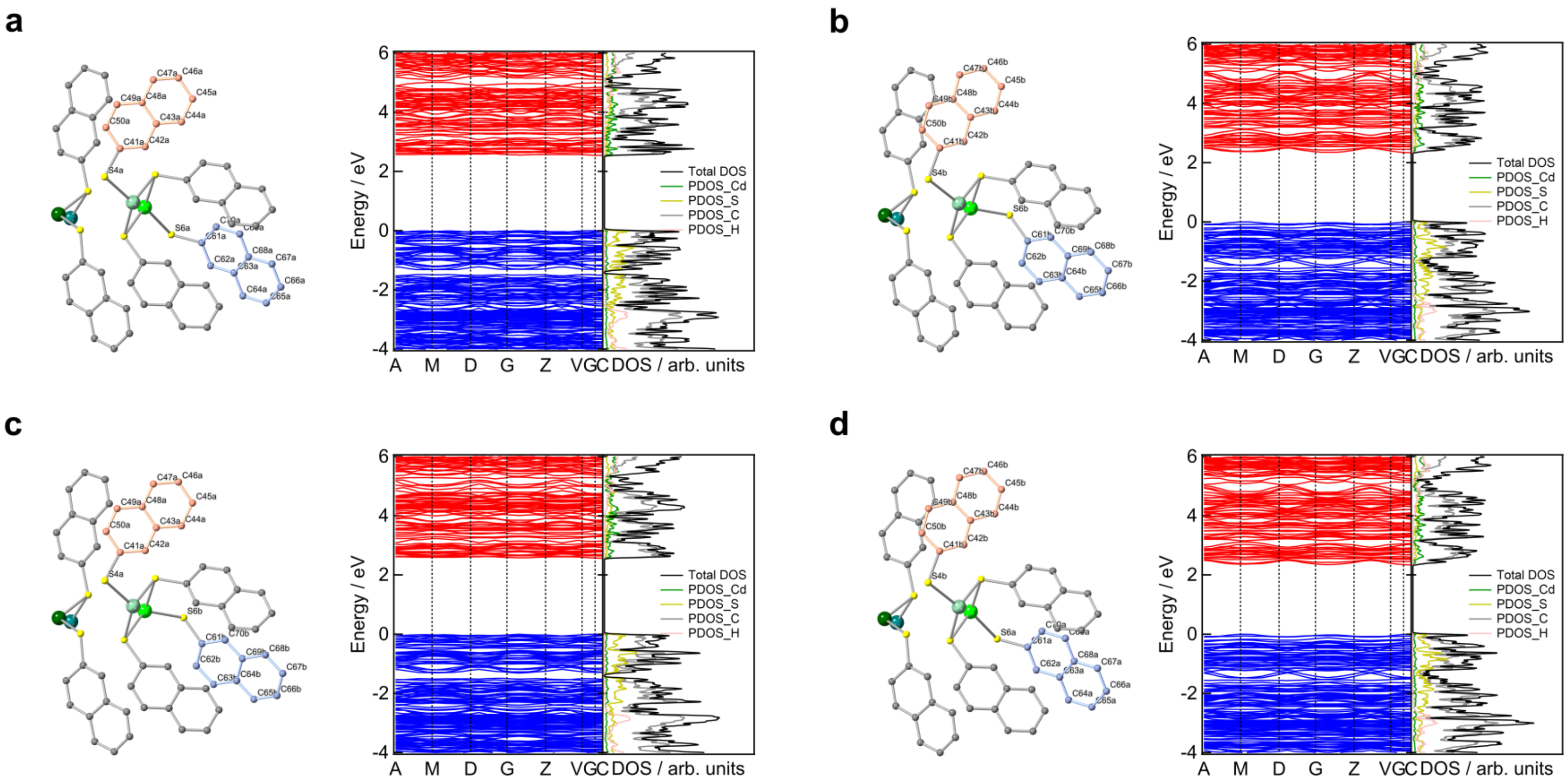

Figure S18. Band structure and PDOS analysis of **KGF-79** calculated using crystallographic models reflecting the disordered 2-SNPh⁻ ligands.

Table S3. PDOS ratio (%) of **KGF-51**, **KGF-71**, and **KGF-79**. The PDOS ratio of **KGF-79** was calculated using structural models generated for the major occupancy configuration observed in the crystal structure.

	**KGF-51**		**KGF-71**		**KGF-79**	
	VBM	CBM	VBM	CBM	VBM	CBM
Cd	3.2	10.3	3.7	11.1	3.9	12.7
S	39.8	3.8	26.4	3.7	30.8	3.9
C	56.5	81.0	69.2	81.6	65.2	83.0
H	0.5	0.7	0	0.5	0.1	0.4

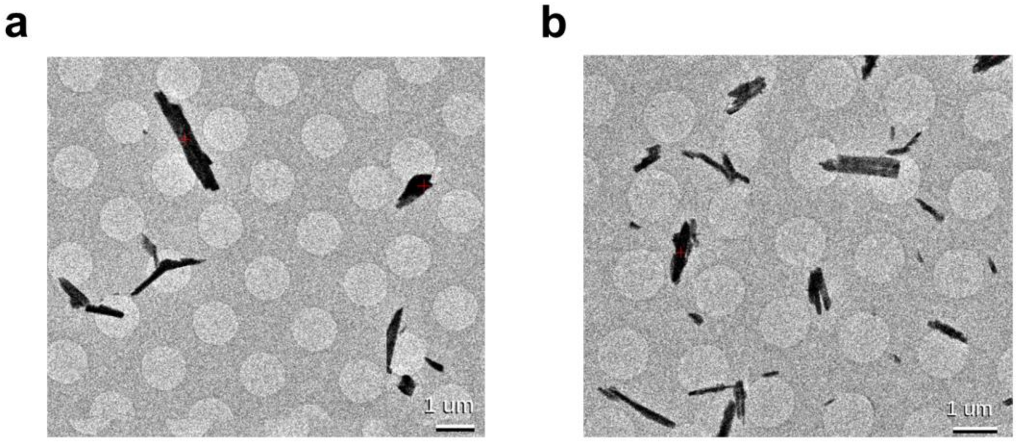

Figure S19. SerialEM montage images of **KGF-79** prepared from (a) **KGF-51** and (b) **KGF-71**. Red crosses indicate measurement positions. Image discontinuities are caused by montage alignment errors.

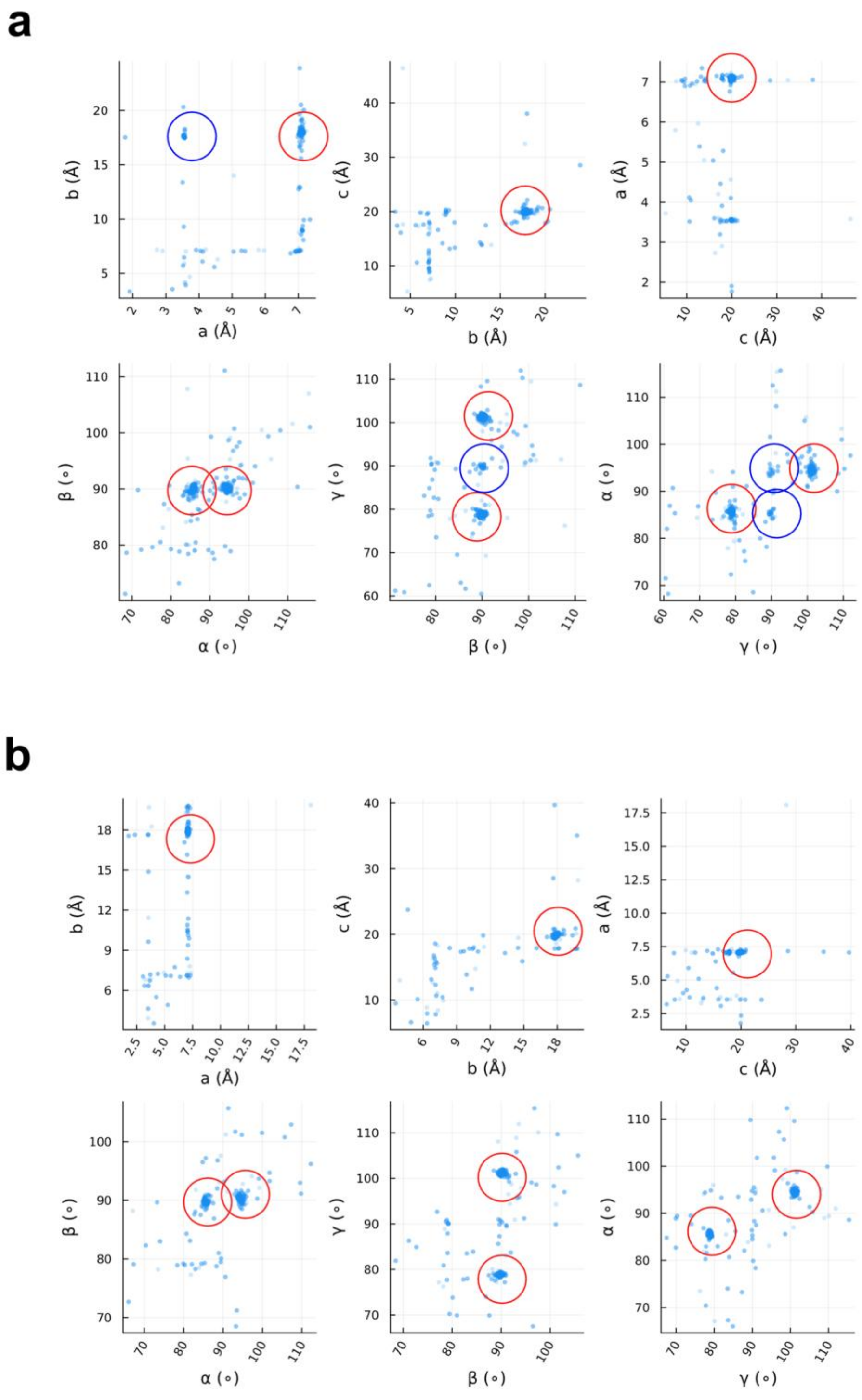

Figure S20. Scatter plots of the unit cell constants of **KGF-79** prepared from (a) **KGF-51** and (b) **KGF-71**. Of the 473 and 303 crystals measured, 353 and 218 crystals, respectively, with resolutions better than 1.2 Å are shown. These are raw results before applying the Bravais lattice constraints and prior cell information and contain misindexed and/or low-quality crystals that were rejected in later steps. The major cluster (circled red) can be reindexed to a *C*-centered monoclinic lattice. Note that angles $90° + x$ and $90° - x$ are equivalent after reindexing. The blue cluster corresponds to misindexed crystals where alternating rows of reflections were missed, leading to half the *a* axis.

Table S4. Crystallographic merging statistics of **KGF-79**.

d_max	d_min	#obs	#uniq	mult.	%comp	<I/sI>	r_pim	cc1/2
6.06	1.70	27242	608	44.81	99.51	40.1	0.029	0.991*
1.70	1.35	31902	578	55.19	100.00	36.4	0.036	0.992*
1.35	1.18	29570	548	53.96	100.00	33.1	0.038	0.992*
1.18	1.08	33747	573	58.90	100.00	30.6	0.036	0.994*
1.08	1.00	30336	536	56.60	100.00	24.3	0.043	0.988*
1.00	0.94	35045	579	60.53	100.00	20.3	0.048	0.976*
0.94	0.89	29453	526	55.99	100.00	15.9	0.055	0.976*
0.89	0.85	35249	574	61.41	100.00	15.7	0.048	0.986*
0.85	0.82	32201	544	59.19	100.00	12.7	0.055	0.968*
0.82	0.79	30825	530	58.16	100.00	11.3	0.059	0.963*
0.79	0.77	35598	567	62.78	100.00	9.3	0.063	0.955*
0.77	0.75	33434	550	60.79	100.00	7.6	0.072	0.941*
0.75	0.73	31652	534	59.27	100.00	5.9	0.084	0.927*
0.73	0.71	30137	517	58.29	100.00	4.6	0.097	0.857*
0.71	0.69	35282	565	62.45	100.00	4.5	0.092	0.866*
0.69	0.68	34572	549	62.97	100.00	3.9	0.107	0.840*
0.68	0.67	32278	538	60.00	100.00	3.1	0.121	0.797*
0.67	0.65	32882	558	58.93	100.00	2.4	0.166	0.654*
0.65	0.64	29535	494	59.79	100.00	1.9	0.178	0.546*
0.64	0.63	36716	569	64.53	100.00	1.8	0.204	0.485*
6.06	0.63	647656	11037	58.68	99.98	14.6	0.040	0.993*
